# A Neoglycoprotein-Immobilized Fluorescent Magnetic Bead Suspension Multiplex Array for Galectin-Binding Studies

**DOI:** 10.3390/molecules26206194

**Published:** 2021-10-14

**Authors:** Libo Zhang, Hai Yu, Yuanyuan Bai, Bijoyananda Mishra, Xiaoxiao Yang, Jing Wang, Evan B. Yu, Riyao Li, Xi Chen

**Affiliations:** 1 Department of Chemistry, University of California, Davis, CA 95616, USA; libzhang@ucdavis.edu (L.Z.); hyu@ucdavis.edu (H.Y.); yuabai@ucdavis.edu (Y.B.); bmishra@ucdavis.edu (B.M.); xiayang@ucdavis.edu (X.Y.); jingwang@qdio.ac.cn (J.W.); evanyu2016@gmail.com (E.B.Y.); lryli@outlook.com (R.L.); 2 Key Laboratory of Experimental Marine Biology, Institute of Oceanology, Chinese Academy of Sciences, Qingdao 266071, China

**Keywords:** carbohydrate-protein conjugate, galectin, glycan-binding protein, lectin, multiplex assay

## Abstract

Carbohydrate-protein conjugates have diverse applications. They have been used clinically as vaccines against bacterial infection and have been developed for high-throughput assays to elucidate the ligand specificities of glycan-binding proteins (GBPs) and antibodies. Here, we report an effective process that combines highly efficient chemoenzymatic synthesis of carbohydrates, production of carbohydrate-bovine serum albumin (glycan-BSA) conjugates using a squarate linker, and convenient immobilization of the resulting neoglycoproteins on carboxylate-coated fluorescent magnetic beads for the development of a suspension multiplex array platform. A glycan-BSA-bead array containing BSA and 50 glycan-BSA conjugates with tuned glycan valency was generated. The binding profiles of six plant lectins with binding preference towards Gal and/or GalNAc, as well as human galectin-3 and galectin-8, were readily obtained. Our results provide useful information to understand the multivalent glycan-binding properties of human galectins. The neoglycoprotein-immobilized fluorescent magnetic bead suspension multiplex array is a robust and flexible platform for rapid analysis of glycan and GBP interactions and will find broad applications.

## 1. Introduction

Glycan-binding proteins (GBPs), anti-glycan antibodies, and many carbohydrate-active enzymes display distinct glycan-binding profiles [1]. Glycan-protein interactions are well known to be involved in diverse biological processes such as infection [2,3], inflammation [4,5], and tumor metastasis [1]. The related studies have attracted increasing interest for both basic and clinical research purposes.

Glycan arrays composed of a large library of glycans immobilized on a type of solid support provide a powerful high-throughput approach to examine glycan-protein interactions. The format allows the capture of multiple copies of glycans on the surface, thus mimicking the multivalent glycan-protein interactions in nature [6]. Various printed slide-based flat surface glycan microarray technologies have been established [7,8,9,10,11,12,13], which have greatly helped to advance our understanding of the glycan-protein interactions. Although successful, the technical demands of using the high precision robotic arraying instrument and high-resolution imaging system for the printed flat surface array technology make it less accessible to non-experts and difficult to be translated to clinical settings [10,14,15]. In comparison, Luminex fluorescent magnetic bead-based suspension arrays [16,17,18,19,20] are rapid, sensitive, flexible, reproducible, easy to use multiplexed assays, and are well suited for analyzing large number of samples [16,18,19,20]. The fluorescent magnetic bead-based array platform is developed from the flow cytometry principle and uses analytes immobilized on beads of 5–7 μm diameter that are identifiable by embedded unique fluorophore combinations [16,18,20]. Numerous Luminex bead-based assays have been approved by the United States Food and Drug Administration (FDA) for clinic diagnosis purposes [18,19,21].

Similar to the printed flat surface glycan microarrays, glycan (the analyte) purity, linker choice (length, structure, function group, etc.), conjugation chemistry, and conditions used will affect the glycan spacing, density, orientation [6,22], and the overall quality of the Luminex fluorescent magnetic bead-based suspension glycan arrays [21], which influence the results of the glycan-protein interaction studies. 

Substantial information regarding GBP-binding specificities has been obtained from microarrays with glycans printed on the flat surface of slides. Some variations regarding GBP-binding specificity have been observed among different arrays [7,8,22,23]. For example, Gildersleeve and coworkers compared their neoglycoprotein-printed slide microarray data for eight plant lectins to those obtained by the Consortium for Functional Glycomics (CFG) using glycan-printed slide microarrays and found that the data from CFG correlated better to those obtained with the low-density neoglycoproteins [22]. In general, generating neoglycoproteins for glycan microarray studies has the significant advantage of being able to determine and control glycan density.

On the other hand, accessing high quantity and structurally defined homogenous glycans by synthesis [24] provides another advantage of reducing the ambiguity of the analyte purity and structure that may be inherited by the materials isolated from natural sources.

**Figure 1:**
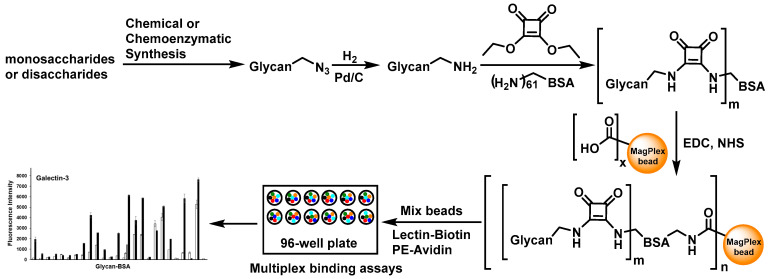
Design and workflow of glycan-BSA neoglycoprotein-immobilized MagPlex bead suspension multiplex array. Abbreviations: EDC and NHS: 1-ethyl-3-(3-dimethylaminopropyl) carbodiimide and *N*-hydroxylsuccinimide, reagents that activate carboxyl groups for conjugation with amines; PE: phycoerythrin, a red protein-pigment complex for fluorescence detection.

Here, we report the combination of highly efficient chemoenzymatic synthesis of oligosaccharides, generation of glycan-bovine serum albumin (glycan-BSA) neoglycoproteins with low and high glycan valencies using a squarate linker, and immobilization of the glycan-BSA on fluorescent magnetic beads (MagPlex beads or Bio-Plex Pro Magnetic COOH Beads) for the development of a multiplex suspension array to study glycan-protein interactions using a Bio-Plex Multiplex Assay System. The strategy and workflow are illustrated in Figure 1. We selected 24 glycosides (with sizes ranging from monosaccharides up to tetrasaccharides) containing D-glucose (Glc), D-galactose (Gal), *N*-acetyl-D-glucosamine (GlcNAc), *N*-acetyl-D-galactosamine (GalNAc) with various glycosidic linkages. By generating and immobilizing these glycan-BSA neoglycoproteins on MagPlex beads, we established a glycan bead array containing a BSA control and 50 glycan-BSA neoglycoprotein conjugates (two glycan-BSA conjugates with low and high glycan valencies, respectively, for each glycan except for LacNAc-BSA conjugate which has four different LacNAc valencies). We established an efficient multiplex binding assay process and characterized the glycan-binding profiles of six plant lectins and two human galectins.

## 2. Results

### 2.1. Glycan Synthesis

**Scheme 1:**
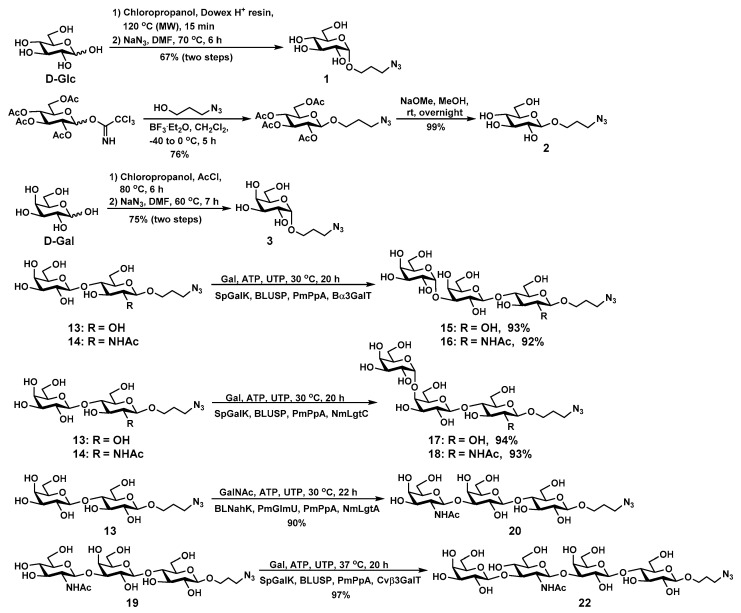
Chemical or enzymatic synthesis of glycans **1**–**3**, **15**–**18**, **20**, and **22**.

There are twenty-four glycosides in the glycan library including eight monosaccharides (**1**–**8**), six disaccharides (**9**–**14**), seven trisaccharides (**15**–**21**), and three tetrasaccharides (**22**–**24**) containing Glc, Gal, GlcNAc, GalNAc with various glycosidic linkages differing on regio- and stereo-specificities (Table S1). In addition to the propylazide (PropN_3_)-tagged glycans terminated with GlcNAc, Gal, GalNAc at the non-reducing end that we synthesized previously [25,26,27,28,29,30], GlcαPropN_3_ (**1**), GlcβPropN_3_ (**2**), GalαPropN_3_ (**3**), Galα3LacβPropN_3_ (**15**), Galα3LacNAcβPropN_3_ (**16**), Galα4LacβPropN_3_, (**17**), Galα4LacNAcβPropN_3_ (**18**), GalNAcβ3LacβPropN_3_ (**20**), and Galβ3GlcNAcβ3LacβPropN_3_ (LNT) (**22**) (Scheme 1) were chemically or enzymatically synthesized. These glycans are important molecular recognition components in glycoproteins, glycolipids, and milk oligosaccharides [31]. For example, glycans carrying Gal or GalNAc residue at the terminal and subterminal positions are the most common receptors for microbial adhesion, which is a process often required for microbial infections [32,33]. Galβ4GlcNAcβOR (Type-2) glycans have been found on the surface of most mammalian cell types; and Galβ3GlcNAcβOR (Type-1) glycans are common components of the O-glycans on glycoproteins and glycolipids presented by the epithelial cells of mammalian gastrointestinal or reproductive tracts [31]. Lac, LNT, LNnT are important milk oligosaccharides [34]. Other glycans such as GA2, GA1, LacNAc, Gb3, iGb3, etc., are components of biologically important glycosphingolipids [29,35].

GlcαPropN_3_ (**1**), GlcβPropN_3_ (**2**), and GalαPropN_3_ (**3**) were synthesized chemically. Briefly, GlcαPropN_3_ (**1**) was synthesized from D-glucose (D-Glc) and chloropropanol in the presence of an ion-exchange resin (H) using microwave-assisted Fischer glycosylation reaction, followed by the conversion of the chloride to azide by treating with sodium azide (NaN_3_) in dimethylformamide (DMF) with a total yield of 67% in two steps. GlcβPropN_3_ (**2**) was synthesized via glycosylation of peracetylated glucose trichloroacetimidate with azidopropanol in the presence of boron trifluoride diethyl etherate (BF_3_·Et_2_O), followed by de-*O*-acetylation using sodium methoxide (NaOMe) in methanol (MeOH). Fischer glycosylation of D-galactose (D-Gal) with chloropropanol in the presence of acetyl chloride (AcCl) produced GalαPropCl, which was converted to GalαPropN_3_ (**3**) by treating with NaN_3_ in DMF with a total yield of 75% in two steps.

Galα3LacβPropN_3_ (**15**), Galα3LacNAcβPropN_3_ (**16**), Galα4LacβPropN_3_, (**17**), Galα4LacNAcβPropN_3_ (**18**), GalNAcβ3LacβPropN_3_ (**20**), and Galβ3GlcNAcβ3LacβPropN_3_ (LNT) (**22**) were synthesized from LacβPropN_3_ (**13**) or LacNAcβPropN_3_ (**14**) using glycosyltransferase-containing one-pot multienzyme (OPME) systems with in situ generation of the sugar nucleotide donor of the glycosyltransferase. Using LacβPropN_3_ (**13**) and LacNAcβPropN_3_ (**14**) as the glycosyltransferase acceptor substrates in an OPME α1–3-galactosylation system containing a recombinant bovine α1–3-galactosyltransferase (Bα3GalT) [36] and uridine 5′-diphosphate galactose (UDP-Gal) biosynthetic enzymes including *Streptococcus pneumoniae* TIGR4 galactokinase (SpGalK) [37], *Bifidobacterium longum* UDP-sugar pyrophosphorylase (BLUSP) [38], and *Pasteurella multocida* inorganic pyrophosphatase (PmPpA) [30], iGb_3_ trisaccharides Galα1–3LacβPropN_3_ (**15**) and Galα1–3LacNAcβPropN_3_ (**16**) were readily synthesized in 93% and 92% yields, respectively. On the other hand, Gb_3_ trisaccharides Galα1–4LacβPropN_3_ (**17**) and Galα1–4LacNAcβPropN_3_ (**18**) were obtained in excellent 94% and 93% yields from LacβPropN_3_ (**13**) and LacNAcβPropN_3_ (**14**), respectively, via an OPME α1–4-galactosylation system containing *N. meningitidis* α1–4-galactosyltransferase (NmLgtC) [39] and the UDP-Gal biosynthetic enzymes including SpGalK, BLUSP, and PmPpA. α-Gal epitope Galα1–3LacNAc is presented abundantly on glycolipids and glycoproteins of non-primate mammals, prosimians, and New World monkeys and has important clinical significance [40]. Galα1–3Lac is the trisaccharide unit of iGb3 glycosphingolipid that has been found on various tissues of many non-primate mammals [41], and iGb3 was reported to act as an endogenous ligand for iNKT cells [42]. P1 trisaccharide Galα1–4LacNAc is the trisaccharide moiety of the P1 antigen, which is one of the key antigens present on red blood cells [43,44]. Galα1–4Lac, also known as the P^k^ antigen, is the trisaccharide unit of Gb3 glycosphingolipid, which is highly expressed on various tumor cells [45].

For the synthesis of GalNAcβ3LacβPropN_3_ (**20**), *N. meningitidis* β1–3-*N*-acetylglucosaminyltransferase (NmLgtA) was utilized. Although NmLgtA has been used commonly for the formation of GlcNAcβ3Lac/LacNAc linkages, it was shown to be able to form GalNAcβ3Lac-type structures efficiently [46]. Trisaccharide GalNAcβ3LacβPropN_3_ (**20**) was synthesized from LacβPropN_3_ (**13**) and *N*-acetyl-D-galactosamine (GalNAc) in 90% yield using a one-pot four-enzyme GalNAc-activation and transfer system containing NmLgtA [35] and uridine 5′-diphosphate *N*-acetylgalactosamine (UDP-GalNAc) biosynthetic enzymes including *Bifidobacterium longum* strain ATCC55813 *N*-acetylhexosamine-1-kinase (BLNahK) [47], *Pasteurella multocida N*-acetylglucosamine uridylyltransferase (PmGlmU) [48], and PmPpA. GalNAcβ3Lac was the oligosaccharide component of ganglioside GAA-7 which showed excellent neuritogenic activity to neuron-like rat adrenal pheochromocytoma (PC12) cells [49,50].

LNTβPropN_3_ (**22**) was synthesized from GlcNAcβ3Galβ4GlcβPropN_3_ (**19**) [28] and Gal in an excellent 97% yield using an OPME Gal-activation and transfer system containing SpGalK, BLUSP, PmPpA, and *Chromobacterium violaceum* β1–3-galactosyltransferase (Cvβ3GalT) [51]. LNT is an oligosaccharide component of lacto-series glycosphingolipids, and also a major human milk oligosaccharide (HMOS) [34,35,52]. 

### 2.2. Glycan-BSA Neoglycoprotein Synthesis

The propylazide aglycone in the structurally defined homogeneous synthetic glycans (**1**–**24**) was reduced by catalytic hydrogenation (Pd/C in water and MeOH) to form glycans with a propylamine aglycone (glycosyl propylamines) [53] which were ready for conjugation.

Diethyl squarate (3,4-diethoxy-3-cyclobutene-1,2-dione), a homobifuncational linker that has been used broadly to conjugate glycans to proteins and other carriers [54,55,56,57], was chosen for conjugating our synthetic glycosyl propylamines to BSA. Glycan-BSA neoglycoproteins were prepared in two steps including squarate derivatization of the glycan followed by conjugation with BSA. 

For the first step, the formation of glycan squarate monoamides, not diamides, was desirable. This was achieved by the higher rate formation of monoamides than diamides in the reactions of a primary or secondary amine and the diethyl squarate [54,58] with a control of the reaction pH and the ratio of the glycan and diethyl squarate. We found that a co-solvent containing a 1:1 ratio of 100 mM NaHCO_3_ and ethanol at pH 9.0 and a diethyl squarate:glycan molar concentration ratio of 2.5:1 were efficient conditions for the formation of the desired glycan squarate monoamide. Diethyl squarate hydrolysis was minimized under these conditions. In general, the reaction was completed in 3 h at room temperature and a yield of around 80% was achieved after purification with a CombiFlash chromatography system equipped with a C18 column. The purified glycan squarate monoamides were analyzed and confirmed by high-resolution mass spectrometry (HRMS) (Table S1).

In the second step for the conjugation of the glycan squarate monoamide to BSA, the ratio of the two reagents was varied in the reaction to test its effect in influencing the glycan valency in the BSA conjugate. We chose a NaHCO_3_ buffer (250 mM) at pH 9.0 [54] and used lactosyl β-propylamine (LacβPropNH_2_, obtained from LacβPropN_3_ **13** by reduction) as a model glycan. The obtained glycan-BSA conjugates were analyzed by MALDI-TOF mass spectrometry and the results were used to calculate the average glycan number per BSA, represented as the glycan valency shown in Table S2. When the molar ratio of LacβPropNH_2_ to BSA used for the conjugation was increased from 20:1 to 100:1, the average number of glycans per BSA (glycan valency) increased from 12 to 36 (Table S2). Therefore, the glycan-BSA valency can be easily controlled by adjusting the glycan to BSA ratio in the squarate coupling process.

### 2.3. Glycan-BSA Immobilization to MagPlex Beads

To determine the amount of glycan-BSA conjugate needed for maximal coating of the MagPlex beads, Lacβ-BSA conjugate with an average of 36 glycans per BSA molecule was used as a model system and *Ricinus communis* agglutinin I (RCA-I), a plant lectin recognizing β-linked lactose [59], was used for detection. As shown in Figure S1, 10 µg (0.12 nmol) of Lacβ-BSA per 25 µL of beads (0.3 × 10^6^ beads) was found to be sufficient for maximal coating as increasing the amount of Lacβ-BSA did not increase the RCA-I binding outcome (Figure S1). The condition was shown to be efficient for the preparation of a glycan-bead library containing 50 different glycan-BSA conjugates and a BSA control (region #14) immobilized on MagPlex beads with 51 different fluorescence regions (one region per glycan-BSA conjugate, see Table S1). The beads of different regions immobilized with different molecules were then pooled together (the same number of beads were used for each bead region immobilized with specific glycan-BSA conjugate or BSA) and used for the assays described below.

### 2.4. Suspension Multiplex Array Assays for Plant Lectins

The bead-based suspension multiplex glycan array platform was used to obtain the glycan-binding profiles of six biotinylated plant lectins including *Artocarpus integrifolia* Lectin (AIA), peanut agglutinin (PNA), *Erythrina cristagalli* Lectin (ECA), *Wisteria floribunda* agglutinin (WFA), *Griffonia simplicifolia* lectin-I (GSL-I), and RCA-I which recognize Gal and/or GalNAc residues. 

**Figure 2:**
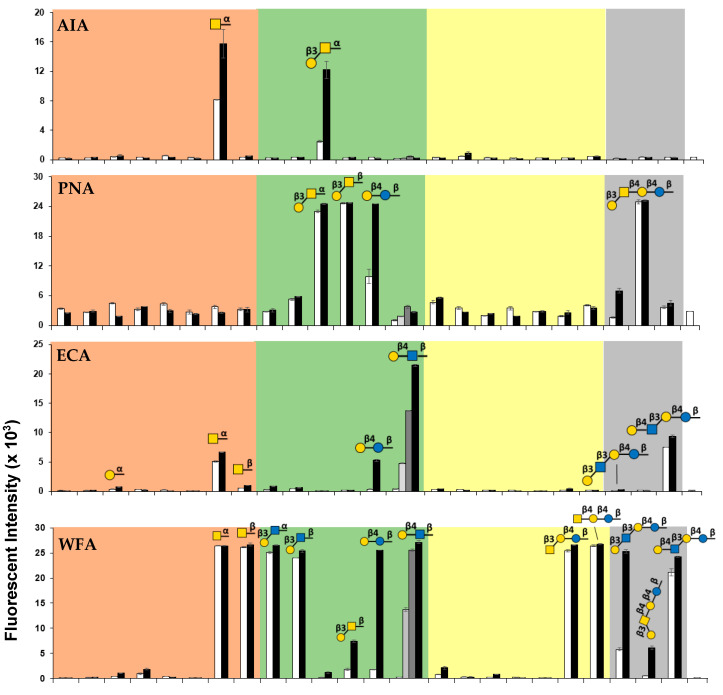

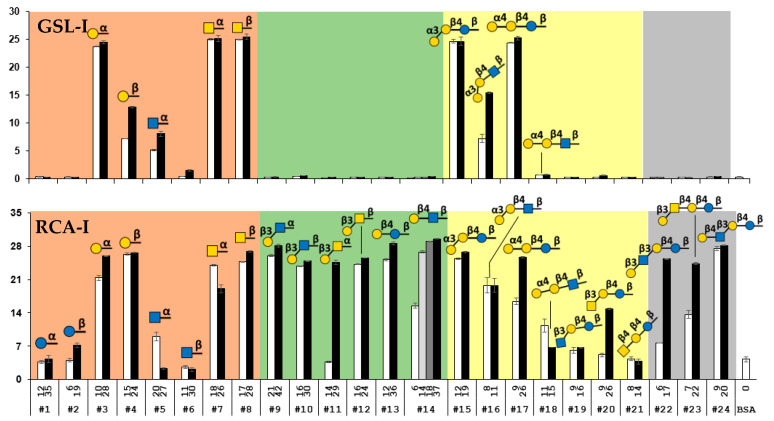
Binding profiles of six plant lectins including AIA, PNA, ECA, WFA GSL-I, and RCA-I studied using the glycan-BSA neoglycoprotein-immobilized bead suspension multiplex array. The glycan numbers (#**1**–#**24**) shown on the bottom X-axis correspond to the glycans shown in Table S1. The values shown above the glycan numbers in the X-axis represent the average numbers of glycans per BSA molecule in the glycan-BSA conjugate. The fluorescence intensity results shown in bars represent the mean ± standard deviation of duplicates.

As shown in Figure 2, the panel of 50 glycan-BSA-beads presenting 24 different glycans clearly differentiates the binding profiles of the six plant lectins tested. In consistent with known general binding preferences of these lectins [22,60,61,62,63,64,65,66], no significant binding of Glcα/βOR (glycans #1 and #2) or GlcNAcβOR (glycan #6) by any of these lectins was observed. Quite interestingly, weak binding of GlcNAcαOR (glycan #5) by GSL-I was observed. In general, among the lectins tested, AIA had the highest specificity while RCA-I had the most promiscuous binding preference towards this library of glycans. PNA, WFA, and GSL-I had almost complete complementary binding specificities except for GalNAcα/βOR (glycans #7 and #8) which was bound by both WFA and GSL-I, and LacβOR (#13) which was recognized by both PNA and WFA. ECA bound only to a subset of glycans that were recognized by WFA. Except for the weak binding of RCA-I to the lower valency Galα4LacNAcβOR (#18), it was not recognized by any other lectins tested, while its counterpart trisaccharide Galα4LacβOR (#17) was recognized by both GSL-I and RCA-I very well. Among the oligosaccharides tested, GlcNAcβ3LacβOR (#19) was the only one that was not recognized by any lectins tested, while both GSL-I and RCA-I bound monosaccharide glycosides Galα/βOR (glycans #3 and #4) and GalNAcα/βOR (glycans #7 and #8) strongly, all disaccharides and tetrasaccharides tested, especially those with the higher valency presentation, were recognized by RCA-I but not by GSL-I.

Our array data showed that AIA bound strongly to GalNAcαOR (#7) and Galβ3GalNAcαOR (#11), while PNA bound strongly to Galβ3GalNAcα/βOR (#11 and #12) and LacβOR (#13) but not GalNAcαOR (#7). These results were consistent with previous reports regarding AIA’s binding specificity to both Tn antigen (GalNAcα) and TF antigen (Galβ3GalNAcα) [62] and PNA’s binding preference towards TF antigen [61,62] and lactose (Lac) [63] but not Tn antigen [62]. The strong binding of PNA to Galβ3GalNAcβ4LacβOR (GA1, #23) was also observed. 

Similar to the previous observation that ECA recognized LacNAc better than Lac [64], our multiplex assay results showed that LacNAcβOR (#14) was the most preferred ligand for ECA while it also bound weakly to LacβOR (#13), LacNAcβ3LacβOR (LNnT, #24), and GalNAcαOR (#7) which was reported previously to inhibit ECA binding to human blood type O erythrocytes [64].

WFA was known to bind selectively to GalNAc- and Gal-terminated glycans [65,67] and was used as an effective diagnostic biomarker for some diseases such as liver fibrosis, liver cirrhosis, prostate, and ovarian cancer, cholangiocarcinoma, and IgA nephropathy [68,69,70]. In our assay, we observed that WFA bound strongly to GalNAc-terminated glycans including GalNAcα/βOR monosaccharide glycosides (#7 and #8), GalNAcβ3LacβOR (#20), and GalNAcβ4LacβOR (GA2, #21), as well as Gal-terminated glycans that was β1–3 or β1–4-linked to GlcNAc or Glc including Galβ3GlcNAcα/βOR (#9 and #10), LacβOR (#13), LacNAcβOR (#14), Galβ3GlcNAcβ3LacβOR (LNT, #22), and LacNAcβ3LacβOR (LNnT, #24). It bound only weakly to high valency Gal-terminated glycans that was β1–3-linked to GalNAc such as Galβ3GalNAcβOR (#12) and Galβ3GalNAcβ4LacβOR (GA1, #23).

GSL-I has been used to identify glycans terminated with α-linked Gal or GalNAc on the cell surface [66] and in tissues [71]. In our assay, we observed strong binding of GSL-I to Galα/βOR (#3 and #4) and GalNAcα/βOR (#7 and #8) monosaccharide glycosides although the binding signal to GalβOR (#4) was lower than others. Strong binding of GSL-I to trisaccharides Galα3LacβOR (iGb3, #15) and Galα4LacβOR (Gb3, #17) was observed, while its binding to Galα3LacNAcβOR (#16) was weaker and no binding to Galα4LacNAcβOR (#18) was detected. The results indicate the importance of the underlying distal glycan residue structure for recognition by GSL-I.

RCA-I has been commonly used to validate and cross-validate different glycan arrays [17,22]. Its binding specificity has been investigated extensively. Consistent with previous reports using printed slide flat surface arrays [13,22,60], RCA-I bound strongly to α or β-linked Gal (#3 and #4) and GalNAc (#7 and #8) monosaccharide glycosides as well as β1–4-linked galactosides such as LacβOR (#13), LacNAcβOR (#14), LacNAcβ3LacβOR (LNnT, #24), and α1–4-linked galactoside Galα4LacβOR (Gb3, #17), but not to β1–4-GalNAc-terminated glycan GalNAcβ4LacβOR (GA2, #21) or β1–3-GlcNAc-terminated glycan GlcNAcβ3LacβOR (#19) in our suspension multiplex bead array. Different from no binding of RCA-I to Galβ3GlcNAcβOR on bead array with immobilized glycans [17] or weaker binding of RCA-I to Galβ3GlcNAc-terminated LNT than Galβ4GlcNAc-terminated on a printed glycan slide flat surface array [72], our suspension multiplex neoglycoprotein-immobilized bead array showed that RCA-I bound strongly to these glycans including Galβ3GlcNAc-terminated glycans Galβ3GlcNAcα/βOR (#9 and #10) and Galβ3GlcNAcβ3LacβOR (LNT, #22). These results aligned better with those obtained from neoglycoprotein-printed slide flat surface arrays [22] which showed strong binding of RCA-I to both Galβ3GlcNAcβOR (glycan valency 15) and Galβ4GlcNAcβOR (glycan valency 15) although the binding for the latter was stronger. Our array showed that RCA-I also bound strongly to Galβ3GalNAc-terminated glycans Galβ3GalNAcβ4LacβOR (GA1, #23), Galβ3GalNAcβOR (#12), and high valency Galβ3GalNAcαOR (#11) as well as glycans with α1–3- or α1–4- terminated Gal including Galα3LacβOR (iGb3, #15), Galα3LacNAcβOR (#16), and high valency Galα4LacβOR (#17). Quite interestingly, RCA-I’s binding to Galα4LacNAcβOR (#18) was weaker than that to Galα4LacβOR (#17) which differed from the former glycan only at the C2-group on the third monosaccharide from the non-reducing end. 

### 2.5. Multiplex Suspension Array Assays for Recombinant Human Galectin-3 and 8

Galectins are a class of lectins named after their β-galactoside-binding preference [73]. While all galectins recognize the key Galβ3/4GlcNAc unit in their ligands [74,75], some have evolved their glycan-binding specificity and have enhanced affinity for ‘repeated’ or ‘branched’ glycans to achieve their functions by multivalent interactions [75,76]. The high-affinity interactions between galectins and glycans assembled at the surface of cells and in the extracellular matrix play important roles in triggering signaling response and modulating biological functions [77,78,79]. Not only the glycan structure but also the density and spatial organization of glycan determinants influence the functions of galectins [80].

We chose to investigate the binding preference of human galectin-3 and galectin-8 using our focused neutral glycan-BSA library with the bead-based multiplex assay. Galectin-3, the only member of the chimera-type galectin, is involved in many biological processes, such as controlling cell-cell and cell-matrix interactions, adhesion, proliferation, apoptosis, immunity, and involvement in the pathogenesis of numerous diseases [81,82,83]. Galectin-8, a member of the tandem-repeat-type galectins, also mediates cell-cell and cell-matrix interactions and is widely expressed in many organs and tissues under physiological or pathological conditions such as inflamed synovia, osteoarthritis, and tumors [84].

**Figure 3:**
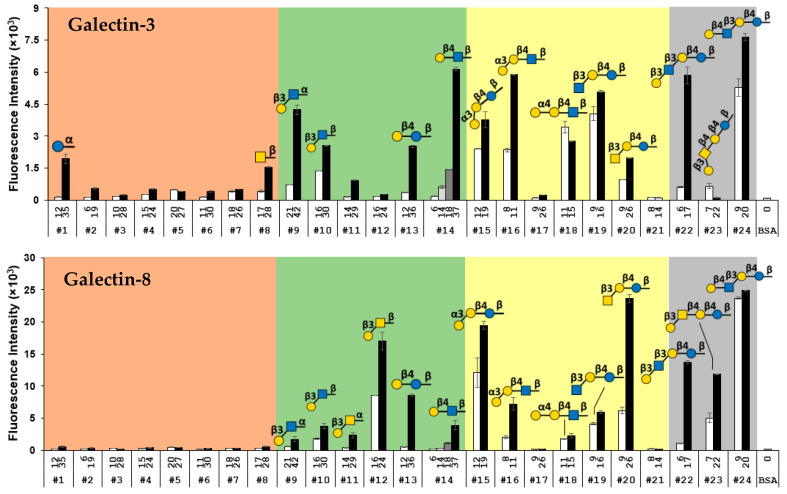
Binding profiles of galectin-3 and galectin-8. The names of glycans are shown in Table S1. The value above each glycan number represents the glycan valency of each glycan-BSA conjugate. The data are presented as the mean ± standard deviation of duplicates.

As shown in Figure 3, galectin-3 and galectin-8 shared some similarities and differences in their binding profiles toward the glycans in the array. Both bound strongly to low and high valency Galβ4GlcNAβ3LacβOR (LNnT, #24) and high valency Galβ3GlcNAcβ3LacβOR (LNT, #22). They also bound to Galα3LacβOR (iGb3, #15) although less strongly. High valency LacβOR (#13) was recognized by both galectins only moderately. Except for weak binding of high valency GlcαOR (glycan #1) and GalNAcβOR (glycan #8) by galectin-3, monosaccharide glycosides tested were not good ligands for either galectin. The results were consistent with a previous report that galectin-8 did not bind to monosaccharide Gal or GlcNAc in a printed slide flat surface array [85]. While minimal binding to Galβ3GalNAαOR (#11) was observed for both galectins, Galα4LacβOR (#17) and GalNAcβ4LacβOR (GA2, #21) were not recognized by either galectin. Their binding towards other glycans varied. Galα4LacNAcβOR (#18), GlcNAcβ3LacβOR (#19), and high valency Galβ3GlcNAcα/βOR (#9 and #10), LacNAcβOR (#14), and Galα3LacNAcβOR (#16) were bound by galectin-3 either moderately or strongly but only weakly by galectin-8. In contrast, galectin-8 bound to high valency Galβ3GalNAcβOR (#12), GalNAcβ3LacβOR (#20), and Galβ3GalNAcβ4LacβOR (GA1, #23) moderately or strongly but they were not bound (#12 and #23) or bound only weakly (#20) by galectin-3. It was worthy to note that LacβOR (#13) was recognized by both galectins moderately but LacNAcβOR (#14) was not recognized by galectin-8 and was bound by galectin-3 only at high valency (average 37 glycans per BSA). 

The binding of both galectins to LacβOR (#13), Galα3LacβOR (iGb3, #15), and GalNAcβ3LacβOR (#20) but neither Galα4LacβOR (Gb3, #17) nor GalNAcβ4LacβOR (GA2 #21) indicated that the 4-OH group of Gal in the lactose motifs was important for recognition, which was consistent with a previous report [75]. 

It was clear from our results that the glycan valency influenced the binding of many glycans by both galectin-3 and galectin-8 significantly. Low valency glycans #1, #8, #9, #13, #14, and #22 lost almost all signals observed from the binding of their high valency counterparts with galectin-3. Similarly, low valency glycans #16 and #22 lost almost all signals from the binding of galectin-8 to their high valency counterparts. The low valency glycan-binding results of galectin-3 aligned well with those obtained from a bead-based glycan array study [17] where no galectin-3 binding to disaccharides including Lac, LacNAc, Galβ3GlcNAc, and Galβ3GalNAc was detected. Similarly, no or very weak binding between galectin-3 and LacNAc and/or Lac was observed in several printed slide flat surface glycan arrays [85,86,87].

## 3. Discussion

Glycopeptides [16,88,89], bacterial lipopolysaccharides [90,91,92], and capsular polysaccharides [93,94,95] have been isolated from natural sources and used to construct glycoconjugate beads for multiplex assays. In comparison, accessing high quantity homogenous synthetic glycans using the highly efficient chemoenzymatic strategy that we report here has the advantage of reducing the ambiguity of the analyte purity and structure. 

Using structurally defined synthetic carbohydrates conjugated to a protein carrier (such as BSA) has the advantage of being able to determine and control the glycan valency that affects downstream glycan-binding assays. BSA is a well-suited protein carrier for generating neoglycoprotein conjugates for glycan-binding assays. It is commercially available, inexpensive, and used broadly in research [96]. It has good water solubility and itself is not glycosylated [97]. The mature BSA protein has 583 amino acid residues including 61 lysine residues presenting primary amino groups [96], most of which are accessible for convenient conjugation reactions. 

On the other hand, diethyl squarate is a well-suited linker reagent. It has been widely applied in carbohydrate chemistry to couple amino group-containing glycans to protein carriers due to its high coupling efficiency under mild reaction conditions [58,98]. The glycan-BSA conjugates produced using the squarate linker, however, have not been used for either printed slide flat surface glycan microarray or bead-based suspension multiplex array. We demonstrated here that the squarate-based conjugation was robust to generate glycan-BSA with different glycan valencies and was well suited for bead-based suspension multiplex glycan array studies. The application of the same linker for the preparation of all neoglycoproteins in the library made the results comparison more straightforward and reliable. It is worth pointing out that the valency of the glycan on the glycan-BSA conjugates could be easily controlled by adjusting the ratio of the glycan and BSA in the conjugation reaction. However, when different glycans were used, the conjugation efficiency could vary, resulting in variations of glycan valency in the obtained products.

Due to the dependence of multivalency presentation of glycans for strong binding by many GBPs and antibodies, preparation of glycan-protein conjugates in the neoglycoprotein form is a well-suited strategy for glycan array studies, not only with the printed slide flat surface platforms but also with the suspension multiplex systems.

Compared to printed slide flat surface glycan microarray platforms, the neoglycoprotein-immobilized fluorescent magnetic bead-based suspension multiplex array is more flexible. As long as additional bead regions are available, other analytes on the beads can be added to an established library. In addition, unlike the production of printed slides which is limited by the printing speed [20], the process for immobilization of analytes on beads is well suited for large-scale production. Furthermore, compared to four spots per glycan commonly used for printed slide-based flat surface glycan microarrays [99], the bead-based suspension multiplex glycan array platform analyzes 50 beads or more per analyte easily, providing a higher scientific rigor. With the additional advantage of ease of handling, glycan-protein conjugate immobilized bead-based suspension multiplex arrays have the potential to become a general strategy for both research and clinical settings [20,21].

## 4. Materials and Methods

### 4.1. Materials and Instruments

Chemicals and reagents were purchased and used as received without further purification unless otherwise specified. 3,4-Diethoxy-3-cyclobutene-1,2-dione (diethyl squarate) was purchased from Ark Pharm (Libertyville, IL, USA). BSA was purchased from Fisher Scientific (Chicago, IL, USA). Microwave-assisted Fischer glycosylation was performed using a Biotage^®^ Initiator+ microwave synthesizer (Biotage, Charlotte, NC, USA). Reversed-Phase C18 Columns (ODS) were purchased from Yamazen Science Inc. USA (Burlingame, CA, USA). ^1^H NMR (800 MHz) and ^13^C NMR (200 MHz) spectra were recorded on a Bruker (Billerica, MA, USA) Avance-800 NMR spectrometer and ^1^H NMR (600 MHz) and ^13^C NMR (150 MHz) spectra were recorded on a Bruker Avance-III HD 600 NMR spectrometer. Thin-layer chromatography (TLC) was performed on silica gel plates (Sorbent Technologies, Norcross, GA, USA) using a UV lamp and anisaldehyde sugar staining for detection. Matrix-assisted laser desorption/ionization (MALDI) mass spectra were obtained using Bruker UltraFlextreme MALDI-TOF (Billerica, MA, USA) at the mass spectrometry facility at the University of California, Davis, CA, USA. High-resolution electrospray ionization (ESI) mass spectra (HSMS) were obtained using the Thermo Fisher Scientific (Waltham, MA, USA) Q Exactive HF Orbitrap Mass Spectrometer at the Mass Spectrometry Facility in the University of California, Davis, USA. Slide-A-Lyzer™ MINI Dialysis Device, 10 k MWCO were purchased from Fisher Scientific (Chicago, IL, USA). IKA MTS 2/4 orbital shaker (IKA Works, Inc., Wilmington, NC, USA) was used for plate shaking. Bio-Plex 200 system, Bio-Plex Handheld Magnetic Washer, Bio-Plex Pro^TM^ Flat Bottom Plates, 16-Tube SureBeadsTM Magnetic Rack, Sheath Fluid, and Bio-Plex Pro^TM^ streptavidin-PE were purchased from Bio-Rad (Hercules, CA, USA). Proteins were purified using Bio-Scale^TM^ Mini Profinity^TM^ TMAC (5 mL) cartridges in an NGC QUEST 10 Chromatography System from Bio-Rad.

### 4.2. Magnetic Fluorescent Beads

Magplex beads (MagPlexTM-C Microspheres or Bio-Plex Pro Magnetic COOH beads) were purchased from Bio-Rad. The diameter of the beads is 6.5 µm. The beads are internally labeled with a combination of two fluorescent dyes for beads identification by a Bio-Plex system and have carboxyl groups on the surface for conjugation. Fifty-one regions of beads (Table S1) were used in this work.

### 4.3. Plant Lectins, Galectins, and Antibodies

Biotin conjugated Artocarpus integrifolia Lectin (Jackfruit)-Jacalin, (AIA) was purchased from EY Laboratories, Inc. (San Mateo, CA, USA). All other biotin-conjugated lectins including RCA I, PNA, ECA, WFA, GS-I were purchased from Vector Laboratories (Burlingame, CA, USA). Human galectin-2 with N-terminal His_6_-tag (*E. coli* source, Cat. No. NBP14832601) and Human galectin-8 with N terminal His_6_ tag (*E. coli* source, Cat. No. NBP14833505) were purchased from Novus Biologicals (Centennial, CO, USA). Biotinylatedmouse anti-6x-His Tag antibody (Invitrogen™, Cat. No. MA121315BTI) was purchased from Fisher Scientific (Chicago, IL, USA).

### 4.4. Syntheisis of Glycans 

Chemical synthesis of GlcαPropN_3_ (**1**). GlcαPropN_3_ (**1**) was synthesized from D-glucose in two steps. Microwave-assisted Fischer glycosylation [100] of D-glucose with 3-chloropropanol was performed by adding D-glucose (1.0 g, 5.56 mmol), Dowex 50 W × 4–100 (H) resin (1.0 g), and anhydrous 3-chloropropanol (10 mL) to a tube reactor vial (20 mL). The reactor vial was then sealed with a Teflon septum and heated in the microwave reactor at 120 °C for 15 min with continuous stirring. The reaction mixture was then filtered and the filtrate was concentrated in vacuo. The resulting dark brown residue was purified by silica column using MeOH:EtOAc = 1:9 by volume as the eluting solvent to yield the product 3-chloropropyl-α-glucopyranoside (1.03 g, 68%) as a colorless sticky solid. The product was dissolved in anhydrous DMF (15 mL), and NaN_3_ (2.61 g, 5.55 mmol) was added. The reaction mixture was heated at 70 °C for 6 h with continuous stirring and then filtered. The filtrate was concentrated in vacuo, and the residue was purified by silica column using MeOH:EtOAc = 1:9 by volume as the eluting solvent. The fractions containing pure products were combined, concentrated in a rotavap, and dried in vacuo to yield the desired product GlcαPropN_3_ (**1**, 1.04 g, 98%) as a colorless sticky syrup. ^1^H NMR (600 MHz, D_2_O) δ 4.92 (d, *J* = 3.6 Hz, 1H), 3.86 (dd, *J* = 2.4, 12.6 Hz, 1H), 3.85–3.79 (m, 1H), 3.76 (dd, *J* = 5.4, 12.6 Hz, 1H), 3.71 (t, *J* = 9.6 Hz, 1H), 3.69–3.66 (m, 1H), 3.62–3.54 (m, 2H), 3.51–3.44 (m, 2H), 3.41(t, *J* = 9.0 Hz, 1H), 2.05–1.76 (m, 2H). ^13^C NMR (150 MHz, D_2_O) δ 98.16, 73.07, 71.81, 71.29, 69.54, 64.98, 60.51, 48.13, 27.96. HRMS (ESI-Orbitrap) *m*/*z*: [M + Cl]^−^ Calcd for C_9_H_17_N_3_O_6_Cl 298.0806; found 298.0807. 

Chemical synthesis of GlcβPropN_3_ (**2**). Peracetylated glucose trichloroacetimidate [101] (0.92 g, 1.87 mmol) was dissolved in CH_2_Cl_2_ (30 mL) containing 4 Å molecular sieves (2 g). 3-Azidopropanol (0.57 g, 5.60 mmol) was added, and the reaction mixture was cooled down to −40 °C. After the mixture was stirred for 30 min, TMSOTf (0.2 eq.) was added dropwise. The mixture was allowed to warm up to 0 °C and incubated for 5 h with stirring using a magnetic bar. The mixture was filtered over Celite and concentrated. The crude material was purified by flash silica gel column (Hexane:EtOAc = 1:2 by volume) to yield preacetylated GlcβPropN_3_ (0.61 g, 76%). Peracetylated GlcβPropN_3_ was dissolved in dry methanol (30 mL) containing a catalytic amount of sodium methoxide. The resulted mixture was stirred at room temperature for overnight, neutralized with Dowex 50 W × 4–100 (H) resin, filtered, and concentrated to yield GlcβPropN_3_ (**2**, syrup, 1.32 g, 99%). ^1^H NMR (800 MHz, D_2_O) δ 4.45 (d, *J* = 8.0 Hz, 1H), 3.99 (dt, *J* = 5.6, 9.6 Hz, 1H), 3.91 (dd, *J* = 2.4, 12.8 Hz, 1H), 3.75 (dt, *J* = 6.4, 10.4 Hz, 1H), 3.71 (dd, *J* = 6.4, 12.8 Hz, 1H), 3.49–3.44 (m, 4H), 3.37 (t, *J* = 9.6 Hz, 1H), 3.26 (t, *J* = 8.8 Hz, 1H), 1.92–1.90 (m, 2H). ^13^C NMR (200 MHz, D_2_O) δ 102.25, 75.89, 75.71, 73.09, 69.63, 67.29, 60.72, 47.86, 28.21. HRMS (ESI) *m*/*z*: [M + Na]^+^ Calcd for C_9_H_17_N_3_O_6_Na 286.1015; found 286.1017. 

Chemical synthesis of GalαPropN_3_ (**3**). To a solution of D-galactose (1.0 g, 5.55 mmol) in 3-chloropropanol (15 mL) acetyl chloride (0.53 g, 6.73 mmol) was added dropwise at 0 °C. The reaction mixture was heated at 80 °C for 6 h. The solution was concentrated and the residue was purified by silica gel chromatography to yield GalαPropCl (1.15 g, 78%). The obtained GalαPropCl (1.0 g, 3.89 mmol) was dissolved in DMF (5 mL), and NaN_3_ (0.304 g, 4.67 mmol) was added. The reaction mixture was heated at 60 °C for 7 h. The reaction mixture was then concentrated and the residue was purified using a short silica gel column with EtOAc:MeOH = 5:1 as the mobile phase. The fractions containing pure products were combined, concentrated in a rotavap, and dried in vacuo to yield GalαPropN_3_ (**3**, syrup, 0.96 g, 94%) as a syrup. ^1^H NMR (800 MHz, D_2_O) δ 4.93 (d, 1H, *J* = 4.0 Hz, H-1), 3.96 (d, 1H, *J* = 4.0 Hz), 3.92 (t, 1H, *J* = 6.4 Hz), 3.84 (dd, 1H, *J* = 3.2, 10.4 Hz), 3.82–3.71 (m, 4H), 3.59–3.42 (m, 3H), 1.95–1.86 (m, 2H); ^13^C NMR (200 MHz, D_2_O) δ 98.24, 70.84, 69.37, 69.15, 68.17, 64.95, 61.08, 48.06, 27.85. HRMS (ESI-Orbitrap) *m*/*z*: [M + Na]^+^ Calcd for C_9_H_17_N_3_O_6_Na 286.1015; found 286.1012.

Enzymatic synthesis of trisaccharides **15** and **16**. A reaction mixture of LacβPropN_3_ or LacNAcβPropN_3_ (20 mM, 1 eq.), Gal (1.5 eq.) in Tris-HCl buffer (100 mM, pH 7.5) containing ATP (1.7 eq.), UTP (1.7 eq.), MgCl_2_ (20 mM), SpGalK (0.2 mg/mL), BLUSP (0.2 mg/mL), Bα1–3GalT (0.3 mg/mL), and PmPpA (0.15 mg/mL) was incubated at 30 °C for 20 h in an incubator shaker with agitation at 100 rpm. The product formation was monitored by LC-MS. When an optimal yield was achieved, the reaction was quenched by adding the same volume of pre-chilled ethanol and the resulting mixture was incubated at 4 °C for 30 min. The precipitates were removed by centrifugation and the supernatant was concentrated. The reaction mixture was purified using a 51 g ODS-SM column (50 µM, 120 Å, Yamazen) on a CombiFlash^®^ Rf 200i system and eluted with gradient acetonitrile in water (0 to 100%). The fractions containing pure products were combined, concentrated in a rotavap, and lyophilized to obtain the desired product Galα3Galβ4GlcβPropN_3_ (**15**, when LacβPropN_3_ was the acceptor) or Galα3Galβ4GlcNAcβPropN_3_ (**16**, when LacNAcβPropN_3_ was the acceptor).

Galα3Galβ4GlcβPropN_3_ (**15**). White amorphous powder, 63 mg, 93%. ^1^H NMR (800 MHz, D_2_O) δ 5.14 (d, *J* = 3.9 Hz, 1H), 4.52 (d, *J* = 8.0 Hz, 1H), 4.49 (d, *J* = 8.0 Hz, 1H), 4.21–4.14 (m, 2H), 4.07–3.51 (m, 17H), 3.46 (t, *J* = 6.4 Hz, 2H), 3.25 (t, *J* = 8.0 Hz, 1H), 1.93–1.89 (m, 2H). ^13^C NMR (200 MHz, D_2_O) δ 102.82, 102.11, 95.40, 78.60, 77.16, 75.02, 74.72, 74.42, 72.76, 70.81, 69.55, 69.26, 69.10, 68.18, 67.35, 64.78, 60.96, 60.89, 60.12, 47.85, 28.21. HRMS (ESI-Orbitrap) *m*/*z*: [M + Na]^+^ Calcd for C_21_H_37_N_3_O_16_Na 610.2072; found 610.2074.

Galα3Galβ4GlcNAcβPropN_3_ (**16**). White amorphous powder, 62 mg, 92%. ^1^H NMR (600 MHz, D_2_O) δ 5.15 (d, *J* = 4.2 Hz, 1H), 4.55 (d, *J* = 8.0, 1H), 4.53 (d, *J* = 8.0, 1H), 4.25–4.12 (m, 2H), 4.07–3.90 (m, 4H), 3.89–3.52 (m, 14H), 3.41–3.33 (m, 2H), 2.05 (s, 3H), 1.87–1.83 (m, 2H). ^13^C NMR (150 MHz, D_2_O) δ 174.42, 102.79, 101.09, 95.41, 78.73, 77.18, 75.02, 74.70, 72.41, 70.81, 69.57, 69.27, 69.11, 68.18, 67.10, 64.79, 60.97, 60.91, 60.13, 55.05, 47.77, 28.10, 22.18. HRMS (ESI-Orbitrap) *m*/*z*: [M + Na]^+^ Calcd for C_23_H_40_N_4_O_16_Na 651.2337; found 651.2331.

Enzymatic synthesis of trisaccharides **17** and **18**. A reaction mixture of LacβPropN_3_ (**13**) or LacNAcβPropN_3_ (**14**) (20 mM, 1 eq.) in Tris-HCl buffer (100 mM, pH 7.5) containing Gal (1.5 eq.), ATP (1.7 eq.), UTP (1.7 eq.), MgCl_2_ (20 mM), SpGalK (0.2 mg/mL), BLUSP (0.2 mg/mL), NmLgtC (0.25 mg/mL), and PmPpA (0.15 mg/mL) was incubated at 30 °C for 20 h in an incubator shaker with agitation at 100 rpm. The product formation was monitored by LC-MS. When an optimal yield was achieved, the reaction was quenched by adding the same volume of pre-chilled ethanol and the mixture was incubated at 4 °C for 30 min. The precipitates were removed by centrifugation and the supernatant was concentrated. The reaction mixture was purified using a 51 g ODS-SM column (50 μM, 120 Å, Yamazen) on a CombiFlash^®^ Rf 200i system and eluted using gradient acetonitrile in water (0 to 100%). The fractions containing pure products were combined, concentrated in a rotavap, and lyophilized to obtain the desired product Galα4Galβ4GlcβPropN_3_ (**17**, when LacβPropN_3_ was the acceptor) or Galα4Galβ4GlcNAcβPropN_3_ (**18**, when LacNAcβPropN_3_ was the acceptor).

Galα4Galβ4GlcβPropN_3_ (**17**). White amorphous powder, 65 mg, 94%. ^1^H NMR (800 MHz, D_2_O) δ 4.94 (d, *J* = 4.0 Hz, 1H), 4.50 (d, *J* = 8.0 Hz, 1H), 4.48 (d, *J* = 8.0 Hz, 1H), 4.34 (t, *J* = 7.2 Hz, 1H), 4.05–3.94 (m, 4H), 3.95–3.86 (m, 2H), 3.86–3.53 (m, 13H), 3.45 (t, *J* = 6.4 Hz, 2H), 3.30 (t, *J* = 8.0 Hz, 1H), 1.93–1.89 (m, 2H). ^13^C NMR (200 MHz, D_2_O) δ 103.24, 102.06, 100.29, 78.63, 77.33, 75.40, 74.78, 74.40, 72.87, 72.14, 70.89, 70.79, 69.10, 68.91, 68.53, 67.33, 60.48, 60.34, 60.01, 47.85, 28.21. HRMS (ESI-Orbitrap) *m*/*z*: [M + Na]^+^ Calcd for C_21_H_37_N_3_O_16_Na 610.2072; found 610.2071.

Galα4Galβ4GlcNAcβPropN_3_ (**18**). White amorphous powder, 63 mg, 93%. ^1^H NMR (600 MHz, D_2_O) δ 4.95 (d, *J* = 4.0 Hz, 1H), 4.53 (d, *J* = 8.0 Hz, 2H), 4.36 (t, *J* = 6.4 Hz, 1H), 4.08–3.94 (m, 4H), 3.95–3.63 (m, 14H), 3.62–3.52 (m, 2H), 3.42–3.35 (m, 2H), 2.05 (s, 3H), 1.89–1.80 (m, 2H). ^13^C NMR (150 MHz, D_2_O) δ 174.49, 103.24, 101.04, 100.26, 78.77, 77.26, 75.40, 74.77, 72.39, 72.13, 70.89, 70.78, 69.12, 68.91, 68.52, 67.11, 60.48, 60.33, 60.01, 55.24, 47.76, 28.09, 22.15. HRMS (ESI-Orbitrap) *m*/*z*: [M + Na]^+^ Calcd for C_23_H_40_N_4_O_16_Na 651.2337; found 651.2330.

Enzymatic Synthesis GalNAcβ3Galβ4GlcβPropN_3_ (**20**). A reaction mixture of LacβPropN_3_ (50 mg, 1.17 mmol, 10 mM), GalNAc (1.5 equiv., 1.76 mmol), ATP (1.5 equiv., 1.76 mmol), and UTP (1.5 equiv., 1.76 mmol) in a Tris-HCl buffer (100 mM, pH 7.5) containing MgCl_2_ (20 mM), NmLgtA (0.5 mg/mL), BLNahK (0.3 mg/mL), PmGlmU (0.3 mg/mL), and PmPpA (0.15 mg/mL) was incubated at 30 °C for 22 h. The reaction progress was monitored using TLC (ethyl acetate:methanol:water = 5:2.4:1, by volume) and mass spectrometry. The reaction mixture was quenched by adding the same volume of pre-chilled ethanol and incubated at 4 °C for 30 min. The mixture was centrifuged, and the supernatant was concentrated. The reaction mixture was purified using a 51 g ODS-SM column (50 μM, 120 Å, Yamazen) on a CombiFlash^®^ Rf 200i system and eluted with a gradient of acetonitrile in water (0 to 100%). The fractions containing pure products were combined, concentrated in a rotavap, and lyophilized to obtain GalNAcβ3Galβ4GlcβPropN_3_ (**20**, white amorphous powder, 67 mg, 90%). ^1^H NMR (800 MHz, D_2_O) δ 4.53 (d, *J* = 8.4 Hz, 1H), 4.40 (d, *J* = 8.0 Hz, 1H), 4.35 (d, *J* = 7.9 Hz, 1H), 4.07 (d, *J* = 3.3 Hz, 1H), 3.95–3.84 (m, 4H), 3.73–3.62 (m, 9H), 3.60–3.48 (m, 5H), 3.38 (t, *J* = 6.7 Hz, 2H), 3.25–3.20 (m, 1H), 1.95 (s, 3H), 1.83 (pd, *J* = 6.6, 1.6 Hz, 2H). ^13^C NMR (200 MHz, D_2_O) δ 175.13, 103.29, 102.93, 102.08, 81.74, 78.33, 74.95, 74.85, 74.75, 74.33, 72.76, 70.71, 70.04, 68.45, 67.72, 67.34, 60.96, 60.92, 60.05, 52.54, 47.84, 28.20, 22.21. HRMS (ESI-Orbitrap) *m*/*z*: [M + Na]^+^ Calcd for C_23_H_40_N_4_NaO_16_ 651.2337; found 651.2307.

Enzymatic Synthesis of Galβ3GlcNAcβ3Galβ4GlcβPropN_3_ (LNTβPropN_3_, **22**). A reaction mixture (10 mL) of GlcNAcβ3Galβ4GlcβPropN_3_ (100 mg, 0.16 mmol), Gal (37 mg, 0.21 mmol), ATP (132 mg, 0.24 mmol), and UTP (130 mg, 0.24 mmol) in Tris-HCl buffer (100 mM, pH 7.5) containing MgCl_2_ (20 mM), SpGalK (0.2 mg/mL), BLUSP (0.2 mg/mL), PmPpA (0.15 mg/mL), and Cvβ3GalT (0.15 mg/mL) was incubated at 37 °C in an incubator shaker for 24 h with shaking (100 rpm). The product formation was monitored by TLC (n-PrOH:H_2_O:NH_4_OH = 5:2:1, by volume, detected with p-anisaldehyde sugar stain) and mass spectrometry (MS). When an optimal yield was achieved, pre-chilled ethanol (10 mL) was added and the mixture was incubated at 4 °C for 30 min. The precipitates were removed by centrifugation and the supernatant was concentrated and purified by a Bio-Gel P-2 gel column (2.5 cm × 80 cm, water was the eluent). The factions containing pure products were combined, concentrated in a rotavap, and lyophilized to obtain LNTβPropN_3_ (**22**, white amorphous powder, 122 mg, 97%). ^1^H NMR (800 MHz, D_2_O) δ 4.73 (d, *J* = 8.8 Hz, 1H), 4.48 (d, *J* = 8.0 Hz, 1H), 4.43 (d, *J* = 8.0 Hz, 2H), 4.15 (d, *J* = 4.0 Hz, 1H), 4.00–3.97 (m, 2H), 3.91–3.69 (m, 15H), 3.65–3.45 (m, 9H), 3.30 (d, *J* = 8.0 Hz,1H), 2.02 (s, 3H), 1.92–1.89 (m, 2H); ^13^C NMR (200 MHz, D_2_O) δ 174.95, 103.47, 102.91, 102.54, 102.09, 82.04, 81.96, 78.35, 75.26, 75.16, 74.87, 74.75, 74.34, 72.76, 72.44, 70.65, 69.97, 68.51, 68.43, 68.29, 67.35, 61.01, 60.93, 60.47, 60.04, 54.68, 47.85, 28.21, 22.20. HRMS (ESI-Orbitrap) *m*/*z*: [M + Na]^+^ Calcd for C_29_H_50_N_4_O_21_Na 813.2865; found 813.2868.

### 4.5. General Procedures for Converting Propylazide-Tagged Glycans to Glycosyl Propylamines

To a stirred solution of a propylazide-tagged glycan (selected from **1**–**24**, 5–10 mg) in water-methanol solution (3 mL, 1:2 by volume), a catalytic amount of 10% palladium on charcoal was added to a 50 mL round bottom flask. The mixture was stirred under a hydrogen environment for 2–5 h. The solution was passed through a syringe filter. Methanol was removed from the filtrate by blowing air inside a fume hood. The residue was lyophilized and used directly for coupling with diethyl squarate without purification.

### 4.6. General Procedures for Preparing Glycan-Squarate Conjugates

Glycosyl propylamine (2–10 mg, 10 mM) was dissolved in NaHCO_3_ (100 mM, pH 9.0) and ethanol (1:1, by volume) cosolvent. Two and a half equiv. of diethyl squarate was added. Diethyl squarate was reported to have the possibility of causing skin allergy [102]. Wear gloves and handle with caution. The mixture was incubated at room temperature with shaking at 850 rpm on an IKA MTS 2/4 orbital shaker. The pH was monitored and adjusted to 9–10 using a NaOH (1 M) solution. The reaction was monitored by TLC (EtOAc/MeOH/H_2_O = 4:2.5:0.5 by volume for monosaccharides and 3:2:1 by volume for disaccharides, trisaccharides, and tetrasaccharides). When the glycan propylamine starting material was completely consumed as detected by TLC (usually takes 3 h), the mixture was concentrated and purified by CombiFlash equipped with a C18 cartridge (13 g). Gradient elution with acetonitrile in water (0 to 100%) was used for the purification. The purified monoamides were collected and characterized by HRMS. 

### 4.7. Coupling Glycan-Squarate to BSA

BSA was dissolved in NaHCO_3_ solution (0.25 mM, pH 9.0) at a concentration of 2 or 4 mg/mL. The same volume of a glycan squarate monoamide (20–100 equiv.) in the same buffer was added. The reaction mixtures were incubated at room temperature for 60 h with shaking at 850 rpm on an IKA MTS 2/4 orbital shaker. The glycan-BSA neoglycoprotein conjugates were dialyzed against 6 mM NaCl using Slide-A-Lyzer™ MINI Dialysis (10 k MWCO) devices at 4 °C for overnight and analyzed by MALDI-TOF mass spectrometry.

### 4.8. Immobilization of Glycan-BSA Neoglycoprotein Conjugates to MagPlex Beads

Bio-Plex Magnetic COOH beads of a specific fluorescence region were used for immobilizing each glycan-BSA neoglycoprotein conjugate. The beads were protected from light as much as possible by covering the tubes with aluminum foil during the whole conjugation procedure. Briefly, the stock vial of beads (1.25 × 10^7^ beads/mL) was vortexed vigorously for 30 s, followed by sonication for 15 s, and 50 µL was aliquoted into a 1.5 mL microcentrifuge tube. The tubes with beads were placed on a SureBeads™ 16-tube magnetic rack for more than 1 min, the storage solution was discarded. The beads were washed once with deionized water (100 µL) and once with MES buffer (100 µL, 0.1 M, pH 6.0) with the help of a SureBeads™ 16-tube magnetic rack. The beads were resuspended in MES buffer (80 µL, 0.1 M, pH 6.0), NHS (10 µL of 50 mg/mL, the final concentration was 91 mM) and EDC (10 µL of 50 mg/mL, the final concentration was 68 mM) was added. The samples were incubated at room temperature for 30 min with shaking at 850 rpm on an IKA MTS 2/4 orbital shaker. Beads were washed three times using MES buffer (100 µL, 0.1 M, pH 6.0), resuspended in phosphate buffer (100 µL, 0.1 M, pH 8.0) containing 20 µg of glycan-BSA neoglycoprotein conjugate, and incubated at room temperature for 2 h. The beads were washed by 100 µL of 0.05% PBST (PBS + 0.05% Tween 20 (*v*/*v*)) twice, quenched by 50 mM ethanoamine in 50 mM Tris-HCl (pH 8.0, 100 µL) at room temperature for 30 min. The beads were then blocked by incubating with 1% BSA (*w*/*v*) in PBS buffer (100 µL) at room temperature for 2 h. The beads were stored in PBS buffer (100 µL) in the dark at 4 °C. As a control, BSA was immobilized a selected region of beads using the same process. 

### 4.9. Prepare Neoglycoprotein-Immobilized MagPlex Beads Library for Binding Assays

The stock vials of beads immobilized with BSA or glycan-BSA neoglycoprotein conjugates were resuspended by vortexing for 30 s, sonicated for 15 s, and added together into a 15 mL centrifuge tube to create an array containing 51 regions of fluorescent magnetic beads (1 for BSA and 50 for glycan-BSA conjugates. Two glycan-BSA conjugates with low and high glycan valencies, respectively, for each glycan except for LacNAc-BSA conjugates which have four different LacNAc valencies. See Table S1 for details). Before binding assays, the mixed beads stock was vortexed and sonicated.

### 4.10. Multiplex Glycan-Binding Assays for Plant Lectins

The assays were carried out in duplicates for each lectin. A total volume of 18 μL of a mixed bead suspension containing around 2250 beads for each bead fluorescence region was transferred into each well of a 96-well Bio-Plex Pro Flat Bottom plate (black) and ddH_2_O (100 μL) was added to each well. A Bio-Plex handheld magnetic washer was used to remove the liquid from the wells by placing the plate on the magnetic washer for more than 1 min to carefully remove all liquid. The beads in each well were then resuspended in 100 µL of TSM buffer (20 mM Tris-HCl, pH 7.4, 150 mM NaCl, 2 mM CaCl_2_, 2 mM MgCl_2_, 0.05% Tween 20) containing 1% BSA and a lectin (50 µg/mL). The plate was covered with aluminum foil and shaken at 850 rpm using an IKA MTS 2/4 Orbital Shaker at room temperature for 1 h. The plate was then washed with 100 µL of TSM buffer twice for each well with the help of the Bio-Plex handheld magnetic washer. The beads of each well were then incubated with TSM buffer (100 µL) containing 1% BSA and freshly prepared 100-fold-diluted streptavidin-PE at room temperature for 1 h with shaking at 850 rpm. The liquid was removed with the help of the Bio-Plex handheld magnetic washer. The beads of each well were washed using TSM buffer (100 µL) twice, then resuspended in 125 µL of TSM buffer without Tween 20 and subjected to analysis by a Bio-Plex 200 system. The bead count setting was 50 (at least 50 beads for each bead fluorescence region were analyzed in each sample).

### 4.11. Multiplex Glycan-Binding Assays for Recombinant Human Galectins

The assays were carried out in duplicate for each galectin. A total volume of 30 μL of a mixed bead suspension with around 3000 beads for each bead fluorescence region was transferred into each well of a 96-well Bio-Plex Pro Flat Bottom plate (black) and ddH_2_O (100 μL) was added to each well. After removing the liquid with the help of the Bio-Plex handheld magnetic washer, the beads in each well were incubated with TSM buffer (100 μL) containing 1% BSA and a recombinant N-terminal His_6_-tagged human galectin (50 µg/mL) at room temperature with shaking (850 rpm) for 1 h and then washed with TSM buffer twice. The beads were then incubated with TSM buffer (100 μL) containing 1% BSA and 3 µg/mL biotinylated anti-His antibody at room temperature for 2 h with shaking (850 rpm) and washed twice using TSM buffer (100 µL). The washed beads were incubated with freshly prepared TSM buffer (100 µL) containing 1% BSA and 100-fold diluted streptavidin-PE at room temperature for 1 h with shaking (850 rpm), washed twice using TSM buffer (100 µL), resuspended in TSM buffer (125 µL) without Tween 20, and analyzed using a Bio-Plex 200 system. The bead count setting was 50 (at least 50 beads for each fluorescence region were analyzed in each sample).

## 5. Conclusions

In conclusion, combining highly efficient chemoenzymatic synthesis, glycan-BSA conjugation with a squarate linker, and fluorescent magnetic bead-based suspension multiplex assay, we established a robust glycan array system comprising a focused library of neutral glycan-BSA conjugates containing glycans of common human monosaccharide building blocks including Glc, Gal, GlcNAc, and GalNAc and the corresponding oligosaccharides with different linkages. The suspension multiplex glycan array was used to differentiate the binding profiles of six Gal/GalNAc-binding plant lectins and to elucidate the binding specificities of human galectin-3 and galectin-8. Compared to printed slide flat surface glycan or glycan-BSA microarray platforms, the bead-based suspension glycan-BSA conjugate arrays have the advantage of flexibility, ease of handling, and the capability of rapid analysis of a large number of samples. We expect that the platform developed in this work can be readily extended to other glycan-binding and inhibition studies for investigating GBPs and antibodies and can be easily translated to clinical diagnostics settings.

## Data Availability

Not applicable.

## Supplementary Materials

The following are available online, Table S1: The list of glycans, glycan-BSA conjugates, and bead region numbers, Table S2: Glycan valency dependence on the ratio of lactosyl squarate monoamide and BSA, Figure S1: RCA-I binding results using MagPlex beads immobilized with different amounts of Lacβ-BSA, Figure S2: MALDI-TOF analysis results of glycan-BSA conjugates, as well as ^1^H and ^13^C{^1^H} NMR spectra of compounds **1**–**3**, **15**–**18**, **20**, and **22**.

## Sample Availability

Samples of compounds are not available from authors.

## References

[1] Taylor M.E., Drickamer K., Schnaar R.L., Etzler M.E., Varki A., Varki A., Cummings R.D., Esko J.D., Stanley P., Hart G.W., Aebi M., Darvill A.G., Kinoshita T., Packer N.H., Prestegard J.H. (2015). Discovery and classification of glycan-binding proteins. Essentials of Glycobiology.

[2] Van Breedam W., Pohlmann S., Favoreel H.W., de Groot R.J., Nauwynck H.J. (2014). Bitter-sweet symphony: Glycan-lectin interactions in virus biology. FEMS Microbiol. Rev..

[3] Imberty A., Varrot A. (2008). Microbial recognition of human cell surface glycoconjugates. Curr. Opin. Struct. Biol..

[4] Marth J.D., Grewal P.K. (2008). Mammalian glycosylation in immunity. Nat. Rev. Immunol..

[5] Arnold J.N., Saldova R., Hamid U.M., Rudd P.M. (2008). Evaluation of the serum N-linked glycome for the diagnosis of cancer and chronic inflammation. Proteomics.

[6] Cohen M., Varki A. (2014). Modulation of glycan recognition by clustered saccharide patches. Int. Rev. Cell Mol. Biol..

[7] Wang L., Cummings R.D., Smith D.F., Huflejt M., Campbell C.T., Gildersleeve J.C., Gerlach J.Q., Kilcoyne M., Joshi L., Serna S. (2014). Cross-platform comparison of glycan microarray formats. Glycobiology.

[8] Padler-Karavani V., Song X., Yu H., Hurtado-Ziola N., Huang S., Muthana S., Chokhawala H.A., Cheng J., Verhagen A., Langereis M.A. (2012). Cross-comparison of protein recognition of sialic acid diversity on two novel sialoglycan microarrays. J. Biol. Chem..

[9] Houseman B.T., Mrksich M. (2002). Carbohydrate arrays for the evaluation of protein binding and enzymatic modification. Chem. Biol..

[10] Blixt O., Head S., Mondala T., Scanlan C., Huflejt M.E., Alvarez R., Bryan M.C., Fazio F., Calarese D., Stevens J. (2004). Printed covalent glycan array for ligand profiling of diverse glycan binding proteins. Proc. Natl. Acad. Sci. USA.

[11] Fukui S., Feizi T., Galustian C., Lawson A.M., Chai W. (2002). Oligosaccharide microarrays for high-throughput detection and specificity assignments of carbohydrate-protein interactions. Nat. Biotechnol..

[12] Chang S.H., Han J.L., Tseng S.Y., Lee H.Y., Lin C.W., Lin Y.C., Jeng W.Y., Wang A.H., Wu C.Y., Wong C.H. (2010). Glycan array on aluminum oxide-coated glass slides through phosphonate chemistry. J. Am. Chem. Soc..

[13] Ratner D.M., Adams E.W., Su J., O’Keefe B.R., Mrksich M., Seeberger P.H. (2004). Probing protein-carbohydrate interactions with microarrays of synthetic oligosaccharides. Chembiochem.

[14] Zhang Y., Gildersleeve J.C. (2012). General procedure for the synthesis of neoglycoproteins and immobilization on epoxide-modified glass slides. Methods Mol. Biol..

[15] Adams E.W., Ueberfeld J., Ratner D.M., O’Keefe B.R., Walt D.R., Seeberger P.H. (2003). Encoded fiber-optic microsphere arrays for probing protein-carbohydrate interactions. Angew. Chem. Int. Ed. Engl..

[16] Yamamoto K., Ito S., Yasukawa F., Konami Y., Matsumoto N. (2005). Measurement of the carbohydrate-binding specificity of lectins by a multiplexed bead-based flow cytometric assay. Anal. Biochem..

[17] Purohit S., Li T., Guan W., Song X., Song J., Tian Y., Li L., Sharma A., Dun B., Mysona D. (2018). Multiplex glycan bead array for high throughput and high content analyses of glycan binding proteins. Nat. Commun..

[18] Kettman J.R., Davies T., Chandler D., Oliver K.G., Fulton R.J. (1998). Classification and properties of 64 multiplexed microsphere sets. Cytometry.

[19] Elshal M.F., McCoy J.P. (2006). Multiplex bead array assays: Performance evaluation and comparison of sensitivity to ELISA. Methods.

[20] Nolan J.P., Sklar L.A. (2002). Suspension array technology: Evolution of the flat-array paradigm. Trends Biotechnol..

[21] Lucas J.L., Tacheny E.A., Ferris A., Galusha M., Srivastava A.K., Ganguly A., Williams P.M., Sachs M.C., Thurin M., Tricoli J.V. (2017). Development and validation of a Luminex assay for detection of a predictive biomarker for PROSTVAC-VF therapy. PLoS ONE.

[22] Temme J.S., Campbell C.T., Gildersleeve J.C. (2019). Factors contributing to variability of glycan microarray binding profiles. Faraday Discuss..

[23] Manimala J.C., Roach T.A., Li Z., Gildersleeve J.C. (2007). High-throughput carbohydrate microarray profiling of 27 antibodies demonstrates widespread specificity problems. Glycobiology.

[24] Oyelaran O., Gildersleeve J.C. (2009). Glycan arrays: Recent advances and future challenges. Curr. Opin. Chem. Biol..

[25] Yu H., Chokhawala H., Karpel R., Yu H., Wu B., Zhang J., Zhang Y., Jia Q., Chen X. (2005). A multifunctional *Pasteurella multocida* sialyltransferase: A powerful tool for the synthesis of sialoside libraries. J. Am. Chem. Soc..

[26] Yu H., Huang S., Chokhawala H., Sun M., Zheng H., Chen X. (2006). Highly efficient chemoenzymatic synthesis of naturally occurring and non-natural alpha-2,6-linked sialosides: A *P. damsela* alpha-2,6-sialyltransferase with extremely flexible donor-substrate specificity. Angew. Chem. Int. Ed. Engl..

[27] Yu H., Thon V., Lau K., Cai L., Chen Y., Mu S., Li Y., Wang P.G., Chen X. (2010). Highly efficient chemoenzymatic synthesis of beta1-3-linked galactosides. Chem. Commun..

[28] Yu H., Zeng J., Li Y., Thon V., Shi B., Chen X. (2016). Effective one-pot multienzyme (OPME) synthesis of monotreme milk oligosaccharides and other sialosides containing 4-O-acetyl sialic acid. Org. Biomol. Chem..

[29] Yang X., Yu H., Yang X., Kooner A.S., Luu B., Chen X. One-pot multienzyme (OPME) chemoenzymatic synthesis of brain ganglioside glycans with human ST3GAL II expressed in *E. coli*.

[30] Lau K., Thon V., Yu H., Ding L., Chen Y., Muthana M.M., Wong D., Huang R., Chen X. (2010). Highly efficient chemoenzymatic synthesis of beta1-4-linked galactosides with promiscuous bacterial beta1-4-galactosyltransferases. Chem. Commun..

[31] Stanley P., Cummings R.D., Varki A., Cummings R.D., Esko J.D., Stanley P., Hart G.W., Aebi M., Darvill A.G., Kinoshita T., Packer N.H., Prestegard J.H. (2015). Structures common to different glycans. Essentials of Glycobiology.

[32] Coombs P.J., Taylor M.E., Drickamer K. (2006). Two categories of mammalian galactose-binding receptors distinguished by glycan array profiling. Glycobiology.

[33] Kim Y.G., Gil G.C., Harvey D.J., Kim B.G. (2008). Structural analysis of alpha-Gal and new non-Gal carbohydrate epitopes from specific pathogen-free miniature pig kidney. Proteomics.

[34] Chen X. (2015). Human milk oligosaccharides (HMOS): Structure, function, and enzyme-catalyzed synthesis. Adv. Carbohydr. Chem. Biochem..

[35] Yu H., Li Y., Zeng J., Thon V., Nguyen D.M., Ly T., Kuang H.Y., Ngo A., Chen X. (2016). Sequential one-pot multienzyme chemoenzymatic synthesis of glycosphingolipid glycans. J. Org. Chem..

[36] Fang J., Li J., Chen X., Zhang Y., Wang J., Guo Z., Zhang W., Yu L., Brew K., Wang P.G. (1998). Highly efficient chemoenzymatic synthesis of α-galactosyl epitopes with a recombinant α(1,3)-galactosyltransferase. J. Am. Chem. Soc..

[37] Chen M., Chen L.L., Zou Y., Xue M., Liang M., Jin L., Guan W.Y., Shen J., Wang W., Wang L. (2011). Wide sugar substrate specificity of galactokinase from *Streptococcus pneumoniae* TIGR4. Carbohydr. Res..

[38] Muthana M.M., Qu J., Li Y., Zhang L., Yu H., Ding L., Malekan H., Chen X. (2012). Efficient one-pot multienzyme synthesis of UDP-sugars using a promiscuous UDP-sugar pyrophosphorylase from *Bifidobacterium longum* (BLUSP). Chem. Commun..

[39] Chen X., Zhang J., Kowal P., Liu Z., Andreana P.R., Lu Y., Wang P.G. (2001). Transferring a biosynthetic cycle into a productive *Escherichia coli* strain:  Large-scale synthesis of galactosides. J. Am. Chem. Soc..

[40] Galili U. (2005). The alpha-gal epitope and the anti-Gal antibody in xenotransplantation and in cancer immunotherapy. Immunol. Cell Biol..

[41] Galili U., Clark M.R., Shohet S.B., Buehler J., Macher B.A. (1987). Evolutionary relationship between the natural anti-Gal antibody and the Gal alpha 1-3Gal epitope in primates. Proc. Natl. Acad. Sci. USA.

[42] Zhou D., Mattner J., Cantu C., Schrantz N., Yin N., Gao Y., Sagiv Y., Hudspeth K., Wu Y.P., Yamashita T. (2004). Lysosomal glycosphingolipid recognition by NKT cells. Science.

[43] Armstrong G.D., Fodor E., Vanmaele R. (1991). Investigation of Shiga-like toxin binding to chemically synthesized oligosaccharide sequences. J. Infect. Dis..

[44] Bruce L.J. (2018). Molecular mechanism of P1 antigen expression. Blood.

[45] Nicolaou K.C., Caulfield T.J., Katoaka H. (1990). Total synthesis of globotriaosylceramide (Gb3) and lysoglobotriaosylceramide (lysoGb3). Carbohydr. Res..

[46] Blixt O., van Die I., Norberg T., van den Eijnden D.H. (1999). High-level expression of the *Neisseria meningitidis lgtA* gene in *Escherichia coli* and characterization of the encoded N-acetylglucosaminyltransferase as a useful catalyst in the synthesis of GlcNAc beta 1→3Gal and GalNAc beta 1→3Gal linkages. Glycobiology.

[47] Li Y., Yu H., Chen Y., Lau K., Cai L., Cao H., Tiwari V.K., Qu J., Thon V., Wang P.G. (2011). Substrate promiscuity of N-acetylhexosamine 1-kinases. Molecules.

[48] Chen Y., Thon V., Li Y., Yu H., Ding L., Lau K., Qu J., Hie L., Chen X. (2011). One-pot three-enzyme synthesis of UDP-GlcNAc derivatives. Chem. Commun..

[49] Tseng H.K., Su Y.Y., Chang T.W., Liu H.C., Li P.J., Chiang P.Y., Lin C.C. (2021). Acceptor-mediated regioselective enzyme catalyzed sialylation: Chemoenzymatic synthesis of GAA-7 ganglioside glycan. Chem. Commun..

[50] Tamai H., Imamura A., Ogawa J., Ando H., Ishida H., Kiso M. (2015). First total synthesis of ganglioside GAA-7 from starfish *Asterias amurensis versicolor*. Eur. J. Org. Chem..

[51] McArthur J.B., Yu H., Chen X. (2019). A bacterial β1-3-galactosyltransferase enables multigram-scale synthesis of human milk lacto-*N*-tetraose (LNT) and its fucosides. ACS Catal..

[52] Bode L. (2012). Human milk oligosaccharides: Every baby needs a sugar mama. Glycobiology.

[53] Yu H., Chokhawala H.A., Varki A., Chen X. (2007). Efficient chemoenzymatic synthesis of biotinylated human serum albumin-sialoglycoside conjugates containing *O*-acetylated sialic acids. Org. Biomol. Chem..

[54] Tietze L.F., Schroter C., Gabius S., Brinck U., Goerlach-Graw A., Gabius H.J. (1991). Conjugation of *p*-aminophenyl glycosides with squaric acid diester to a carrier protein and the use of neoglycoprotein in the histochemical detection of lectins. Bioconjug. Chem..

[55] Blixt O., Norberg T. (1999). Enzymatic glycosylation of reducing oligosaccharides linked to a solid phase or a lipid via a cleavable squarate linker. Carbohydr. Res..

[56] Kamath V.P., Diedrich P., Hindsgaul O. (1996). Use of diethyl squarate for the coupling of oligosaccharide amines to carrier proteins and characterization of the resulting neoglycoproteins by MALDI-TOF mass spectrometry. Glycoconj. J..

[57] Nitz M., Bundle D.R. (2001). Synthesis of di- to hexasaccharide 1,2-linked beta-mannopyranan oligomers, a terminal S-linked tetrasaccharide congener and the corresponding BSA glycoconjugates. J. Org. Chem..

[58] Wurm F.R., Klok H.-A. (2013). Be squared: Expanding the horizon of squaric acid-mediated conjugations. Chem. Soc. Rev..

[59] Cummings R.D., Schnaar R.L., Varki A., Cummings R.D., Esko J.D., Stanley P., Hart G.W., Aebi M., Darvill A.G., Kinoshita T., Packer N.H., Prestegard J.H. (2015). R-type lectins. Essentials of Glycobiology.

[60] Green E.D., Brodbeck R.M., Baenziger J.U. (1987). Lectin affinity high-performance liquid chromatography. Interactions of N-glycanase-released oligosaccharides with *Ricinus communis* agglutinin I and *Ricinus communis* agglutinin II. J. Biol. Chem..

[61] Li N., Chow A.M., Ganesh H.V., Brown I.R., Kerman K. (2013). Quantum dot based fluorometric detection of cancer TF-antigen. Anal. Chem..

[62] Duk M., Lisowska E., Wu J.H., Wu A.M. (1994). The biotin/avidin-mediated microtiter plate lectin assay with the use of chemically modified glycoprotein ligand. Anal. Biochem..

[63] Sharma V., Vijayan M., Surolia A. (1996). Imparting exquisite specificity to peanut agglutinin for the tumor-associated Thomsen-Friedenreich antigen by redesign of its combining site. J. Biol. Chem..

[64] Iglesias J.L., Lis H., Sharon N. (1982). Purification and properties of a D-galactose/N-acetyl-D-galactosamine-specific lectin from *Erythrina cristagalli*. Eur. J. Biochem..

[65] Kurokawa T., Tsuda M., Sugino Y. (1976). Purification and characterization of a lectin from *Wistaria floribunda* seeds. J. Biol. Chem..

[66] Qiu Y., Tian S., Gu L., Hildreth M., Zhou R. (2019). Identification of surface polysaccharides in akinetes, heterocysts and vegetative cells of *Anabaena cylindrica* using fluorescein-labeled lectins. Arch. Microbiol..

[67] Sato T., Tateno H., Kaji H., Chiba Y., Kubota T., Hirabayashi J., Narimatsu H. (2017). Engineering of recombinant *Wisteria floribunda* agglutinin specifically binding to GalNAcβ1,4GlcNAc (LacdiNAc). Glycobiology.

[68] Hartig W., Brauer K., Bruckner G. (1992). *Wisteria floribunda* agglutinin-labelled nets surround parvalbumin-containing neurons. Neuroreport.

[69] Matsuda A., Kuno A., Kawamoto T., Matsuzaki H., Irimura T., Ikehara Y., Zen Y., Nakanuma Y., Yamamoto M., Ohkohchi N. (2010). *Wisteria floribunda* agglutinin-positive mucin 1 is a sensitive biliary marker for human cholangiocarcinoma. Hepatology.

[70] Narimatsu H., Sato T. (2018). *Wisteria floribunda* agglutinin positive glycobiomarkers: A unique lectin as a serum biomarker probe in various diseases. Expert Rev. Proteom..

[71] Schick B., Habermann F., Sinowatz F. (2009). Histochemical detection of glycoconjugates in the canine epididymis. Anat. Histol. Embryol..

[72] Yu Y., Mishra S., Song X., Lasanajak Y., Bradley K.C., Tappert M.M., Air G.M., Steinhauer D.A., Halder S., Cotmore S. (2012). Functional glycomic analysis of human milk glycans reveals the presence of virus receptors and embryonic stem cell biomarkers. J. Biol. Chem..

[73] Barondes S.H., Castronovo V., Cooper D.N., Cummings R.D., Drickamer K., Feizi T., Gitt M.A., Hirabayashi J., Hughes C., Kasai K. (1994). Galectins: A family of animal beta-galactoside-binding lectins. Cell.

[74] Cummings R.D., Liu F.T., Vasta G.R., Varki A., Cummings R.D., Esko J.D., Stanley P., Hart G.W., Aebi M., Darvill A.G., Kinoshita T., Packer N.H., Prestegard J.H. (2015). Galectins. Essentials of Glycobiology.

[75] Hirabayashi J., Hashidate T., Arata Y., Nishi N., Nakamura T., Hirashima M., Urashima T., Oka T., Futai M., Muller W.E. (2002). Oligosaccharide specificity of galectins: A search by frontal affinity chromatography. Biochim. Biophys. Acta.

[76] Fred Brewer C. (2002). Binding and cross-linking properties of galectins. Biochim. Biophys. Acta.

[77] Dam T.K., Gerken T.A., Brewer C.F. (2009). Thermodynamics of multivalent carbohydrate-lectin cross-linking interactions: Importance of entropy in the bind and jump mechanism. Biochemistry.

[78] Mammen M., Choi S.K., Whitesides G.M. (1998). Polyvalent interactions in biological systems: Implications for design and use of multivalent ligands and inhibitors. Angew. Chem. Int. Ed. Engl..

[79] Godula K. (2018). Following sugar patterns in search of galectin function. Proc. Natl. Acad. Sci. USA.

[80] Xiao Q., Ludwig A.K., Romano C., Buzzacchera I., Sherman S.E., Vetro M., Vertesy S., Kaltner H., Reed E.H., Moller M. (2018). Exploring functional pairing between surface glycoconjugates and human galectins using programmable glycodendrimersomes. Proc. Natl. Acad. Sci. USA.

[81] Tan Y., Zheng Y., Xu D., Sun Z., Yang H., Yin Q. (2021). Galectin-3: A key player in microglia-mediated neuroinflammation and Alzheimer’s disease. Cell Biosci..

[82] Blanda V., Bracale U.M., Di Taranto M.D., Fortunato G. (2020). Galectin-3 in cardiovascular diseases. Int. J. Mol. Sci..

[83] Sciacchitano S., Lavra L., Morgante A., Ulivieri A., Magi F., De Francesco G.P., Bellotti C., Salehi L.B., Ricci A. (2018). Galectin-3: One molecule for an alphabet of diseases, from A to Z. Int. J. Mol. Sci..

[84] Tribulatti M.V., Carabelli J., Prato C.A., Campetella O. (2020). Galectin-8 in the onset of the immune response and inflammation. Glycobiology.

[85] Horlacher T., Oberli M.A., Werz D.B., Krock L., Bufali S., Mishra R., Sobek J., Simons K., Hirashima M., Niki T. (2010). Determination of carbohydrate-binding preferences of human galectins with carbohydrate microarrays. Chembiochem.

[86] Song X., Xia B., Stowell S.R., Lasanajak Y., Smith D.F., Cummings R.D. (2009). Novel fluorescent glycan microarray strategy reveals ligands for galectins. Chem. Biol..

[87] Stowell S.R., Arthur C.M., Mehta P., Slanina K.A., Blixt O., Leffler H., Smith D.F., Cummings R.D. (2008). Galectin-1, -2, and -3 exhibit differential recognition of sialylated glycans and blood group antigens. J. Biol. Chem..

[88] Yamamoto K., Yasukawa F., Ito S. (2007). Measurement of the sugar-binding specificity of lectins using multiplexed bead-based suspension arrays. Methods Mol. Biol..

[89] Yamamoto K. (2014). Carbohydrate-binding specificity of lectins using multiplexed glyco-bead array. Methods Mol. Biol..

[90] Iihara H., Niwa T., Shah M.M., Nhung P.H., Song S.X., Hayashi M., Ohkusa K., Itoh Y., Makino S., Ezaki T. (2007). Rapid multiplex immunofluorescent assay to detect antibodies against *Burkholderia pseudomallei* and taxonomically closely related nonfermenters. Jpn. J. Infect. Dis..

[91] Berger S.S., Lauritsen K.T., Boas U., Lind P., Andresen L.O. (2017). Simultaneous detection of antibodies to five *Actinobacillus pleuropneumoniae* serovars using bead-based multiplex analysis. J. Vet. Diagn. Investig..

[92] Kaminski R.W., Clarkson K., Kordis A.A., Oaks E.V. (2013). Multiplexed immunoassay to assess Shigella-specific antibody responses. J. Immunol. Methods.

[93] Gray B.M. (1979). ELISA methodology for polysaccharide antigens: Protein coupling of polysaccharides for adsorption to plastic tubes. J. Immunol. Methods.

[94] Pickering J.W., Martins T.B., Greer R.W., Schroder M.C., Astill M.E., Litwin C.M., Hildreth S.W., Hill H.R. (2002). A multiplexed fluorescent microsphere immunoassay for antibodies to pneumococcal capsular polysaccharides. Am. J. Clin. Pathol..

[95] Pickering J.W., Hill H.R., Chevolot Y. (2012). Measurement of antibodies to pneumococcal polysaccharides with Luminex xMAP microsphere-based liquid arrays. Carbohydrate Microarrays: Methods and Protocols.

[96] Bujacz A., Zielinski K., Sekula B. (2014). Structural studies of bovine, equine, and leporine serum albumin complexes with naproxen. Proteins.

[97] Bundle D.R., Tam P.H., Tran H.A., Paszkiewicz E., Cartmell J., Sadowska J.M., Sarkar S., Joe M., Kitov P.I. (2014). Oligosaccharides and peptide displayed on an amphiphilic polymer enable solid phase assay of hapten specific antibodies. Bioconjug. Chem..

[98] Bocker S., Laaf D., Elling L. (2015). Galectin binding to neo-glycoproteins: LacDiNAc conjugated BSA as ligand for human galectin-3. Biomolecules.

[99] Smith D.F., Song X., Cummings R.D. (2010). Use of glycan microarrays to explore specificity of glycan-binding proteins. Methods Enzymol..

[100] Bornaghi L.F., Poulsen S.-A. (2005). Microwave-accelerated Fischer glycosylation. Tetrahedron Lett..

[101] Cheng H., Cao X., Xian M., Fang L., Cai T.B., Ji J.J., Tunac J.B., Sun D., Wang P.G. (2005). Synthesis and enzyme-specific activation of carbohydrate−geldanamycin conjugates with potent anticancer activity. J. Med. Chem..

[102] Tietze L.F., Arlt M., Beller M., Gl üsenkamp K.-H., Jähde E., Rajewsky M.F. (1991). Anticancer agents, 15. Squaric acid diethyl ester: A new coupling reagent for the formation of drug biopolymer conjugates. Synthesis of squaric acid ester amides and diamides. Chem. Ber..

