# Supplementary material for: A Neoglycoprotein-Immobilized Fluorescent Magnetic Bead Suspension Multiplex Array for Galectin-Binding Studies
Source: Molecules. 2021 Oct 14;26(20):6194. doi: 10.3390/molecules26206194 (PMC8541226; doi:10.3390/molecules26206194)

Supporting Information

A neoglycoprotein-immobilized fluorescent magnetic bead suspension multiplex array for galectin-binding studies

Libo Zhang[1], Hai Yu[1], Yuanyuan Bai[1], Bijoyananda Mishra[1], Xiaoxiao Yang[1], Jing Wang[1,2], Evan B. Yu[1], Riyao Li[1], and Xi Chen[1]*

[1]Department of Chemistry, University of California, Davis, California, United States
[2]Key Laboratory of Experimental Marine Biology, Institute of Oceanology, Chinese Academy of Sciences, Qingdao, China
*Correspondence: xiichen@ucdavis.edu; Tel.: 1-530-754-6037

Table of content

Table S1. The list of glycans, glycan-BSA conjugates, and bead region numbers……..............S2–S3
Reference…………………………………………………………………………………......S3
Table S2. Glycan valency dependence on the ratio of lactosyl squarate monoamide and BSA...…...S4
Figure S1. RCA-I binding results using MagPlex beads immobilized with different amounts of Lacβ-BSA…………………………………………………………………………………....S4
Figure S2. MALDI-TOF analysis results of glycan-BSA conjugates………………………….S5–S7
^{1}H and ^{13}C{^{1}H} NMR spectra of compounds 1–3, **15–18**, **20**, and **22**...................................S8–S16

Table S1. The list of glycans, glycan-BSA conjugates, and bead region numbers.

Compound number	Glycan	Ref.	HRMS of glycan squarate	Average glycan number per BSA	Beads Region number
**1**	GlcαPropN$_3$	This study	m/z: $[M+Na]^+$ Calcd for $C_{15}H_{23}NO_9Na$ 384.1271; found 384.1232	12	64
				35	65
**2**	GlcβPropN$_3$	This study	m/z: $[M+Na]^+$ Calcd for $C_{15}H_{23}NO_9Na$ 384.1271; found 384.1256	6	18
				19	15
**3**	GalαPropN$_3$	This study	m/z: $[M+Na]^+$ Calcd for $C_{15}H_{23}NO_9Na$ 384.1271; found 384.1234	10	58
				28	61
**4**	GalβPropN$_3$	[1]	m/z: $[M+Na]^+$ Calcd for $C_{15}H_{23}NO_9Na$ 384.1271; found 384.1257	15	20
				24	19
**5**	GlcNAcαPropN$_3$	[2]	m/z: $[M+Na]^+$ Calcd for $C_{17}H_{26}N_2O_9Na$ 425.1536; found 425.1517	20	51
				27	47
**6**	GlcNAcβPropN$_3$	[2]	m/z: $[M+Na]^+$ Calcd for $C_{17}H_{26}N_2O_9Na$ 425.1536; found 425.1496	11	30
				30	29
**7**	GalNAcαPropN$_3$	[3]	m/z: $[M+Na]^+$ Calcd for $C_{17}H_{26}N_2O_9Na$ 425.1536; found 425.1524	18	46
				26	45
**8**	GalNAcβPropN$_3$	[3]	m/z: $[M+Na]^+$ Calcd for $C_{17}H_{26}N_2O_9Na$ 425.1536; found 425.1518	17	22
				28	21
**9**	Galβ3GlcNAcαPropN$_3$	[4]	m/z: $[M+Na]^+$ Calcd for $C_{23}H_{36}N_2O_{14}Na$ 587.2064; found 587.2053	21	35
				42	34
**10**	Galβ3GlcNAcβPropN$_3$	[4]	m/z: $[M+Na]^+$ Calcd for $C_{23}H_{36}N_2O_{14}Na$ 587.2064; found 587.2057	16	54
				30	52
**11**	Galβ3GalNAcαPropN$_3$	[4]	m/z: $[M+Na]^+$ Calcd for $C_{23}H_{36}N_2O_{14}Na$ 587.2064; found 587.2060	14	77
				29	78
**12**	Galβ3GalNAcβPropN$_3$	[4]	m/z: $[M+Na]^+$ Calcd for $C_{23}H_{36}N_2O_{14}Na$ 587.2064; found 587.2057	16	42
				24	39
**13**	Galβ4GlcβPropN$_3$ (LacβProN$_3$)	[1, 3]	m/z: $[M+Na]^+$ Calcd for $C_{21}H_{33}NO_{14}Na$ 546.1799; found 546.1753	12	73
				36	74
**14**	Galβ4GlcNAcβPropN$_3$ (LacNAcβProN$_3$)	[2]	m/z: $[M+Na]^+$ Calcd for $C_{23}H_{36}N_2O_{14}Na$ 587.2064; found 587.2044	6	75
				14	76
				18	7
				37	36
**15**	Galα3LacβPropN$_3$ (iGb3)	This study	m/z: $[M+Na]^+$ Calcd for $C_{27}H_{43}NO_{19}Na$ 708.2327; found 708.2319	12	44
				19	43
**16**	Galα3LacNAcβPropN$_3$	This study	m/z: $[M+Na]^+$ Calcd for $C_{29}H_{46}N_2O_{19}Na$ 749.2592; found 749.2559	8	53
				11	55
**17**	Galα4LacβPropN$_3$ (Gb3)	This study	m/z: $[M+Na]^+$ Calcd for $C_{27}H_{43}NO_{19}Na$ 708.2327; found 708.2338	9	67
				26	72
**18**	Galα4LacNAcβPropN$_3$	This study	m/z: $[M+Na]^+$ Calcd for $C_{29}H_{46}N_2O_{19}Na$ 749.2592; found 749.2594	11	38
				15	37

**19**	GlcNAcβ3LacβPropN$_3$	[5]	m/z: $[M+Na]^+$ Calcd for $C_{29}H_{46}N_2O_{19}Na$ 749.2592; found 749.2588	9	26
				16	25
**20**	GalNAcβ3LacβPropN$_3$	This study	m/z: $[M+Na]^+$ Calcd for $C_{29}H_{46}N_2O_{19}Na$ 749.2592; found 749.2586	9	67
				26	72
**21**	GalNAcβ4LacβPropN$_3$ (GA2)	[6]	m/z: $[M+Na]^+$ Calcd for $C_{29}H_{46}N_2O_{19}Na$ 749.2592; found 749.2559	8	9
				14	8
**22**	Galβ3GlcNAcβ3LacβPropN$_3$ (LNT)	This study	m/z: $[M+Na]^+$ Calcd for $C_{35}H_{56}N_2O_{24}Na$ 911.3121; found 911.3041	6	63
				17	62
**23**	Galβ3GalNAcβ4LacβPropN$_3$ (GA1)	[6]	m/z: $[M+Na]^+$ Calcd for $C_{35}H_{56}N_2O_{24}Na$ 911.3121; found 911.3101	7	13
				22	12
**24**	Galβ4GlcNAcβ3LacβPropN$_3$ (LNnT)	[5]	m/z: $[M+Na]^+$ Calcd for $C_{35}H_{56}N_2O_{24}Na$ 911.3121; found 911.3103	9	28
				20	27

References:

1. Yu, H.; Chokhawala, H.; Karpel, R.; Yu, H.; Wu, B.; Zhang, J.; Zhang, Y.; Jia, Q.; Chen, X. A multifunctional *Pasteurella multocida* sialyltransferase: a powerful tool for the synthesis of sialoside libraries. *J. Am. Chem. Soc.* **2005,** *127*, 17618-17619, doi:10.1021/ja0561690.
2. Lau, K.; Thon, V.; Yu, H.; Ding, L.; Chen, Y.; Muthana, M. M.; Wong, D.; Huang, R.; Chen, X. Highly efficient chemoenzymatic synthesis of beta1-4-linked galactosides with promiscuous bacterial beta1-4-galactosyltransferases. *Chem. Commun.* **2010,** *46*, 6066-6068, doi:10.1039/c0cc01381a.
3. Yu, H.; Huang, S.; Chokhawala, H.; Sun, M.; Zheng, H.; Chen, X. Highly efficient chemoenzymatic synthesis of naturally occurring and non-natural alpha-2,6-linked sialosides: a *P. damsela* alpha-2,6-sialyltransferase with extremely flexible donor-substrate specificity. *Angew. Chem. Int. Ed. Engl.* **2006,** *45*, 3938-3944, doi:10.1002/anie.200600572.
4. Yu, H.; Thon, V.; Lau, K.; Cai, L.; Chen, Y.; Mu, S.; Li, Y.; Wang, P. G.; Chen, X. Highly efficient chemoenzymatic synthesis of beta1-3-linked galactosides. *Chem. Commun.* **2010,** *46*, 7507-7509, doi:10.1039/c0cc02850a.
5. Yu, H.; Zeng, J.; Li, Y.; Thon, V.; Shi, B.; Chen, X. Effective one-pot multienzyme (OPME) synthesis of monotreme milk oligosaccharides and other sialosides containing 4-*O*-acetyl sialic acid. *Org. Biomol. Chem.* **2016,** *14*, 8586-8597, doi:10.1039/c6ob01706a.
6. Yang, X.; Yu, H.; Yang, X.; Kooner, A. S.; Luu, B.; Chen, X. One-pot multienzyme (OPME) chemoenzymatic synthesis of brain ganglioside glycans with human ST3GAL II expressed in *E. coli*. Submitted.

Table S2. Glycan valency (average number of glycans per BSA molecule) dependence on the ratio of lactosyl squarate monoamide and BSA. Conditions used: 2 mg/mL BSA was incubated with different ratios (20:1 to 100:1) of lactosyl squarate monoamide at room temperature for 20 h with shaking (850 rpm).

**Lactoside:BSA ratio**	**Glycan valency**
20:1	12
40:1	20
60:1	28
80:1	32
100:1	36

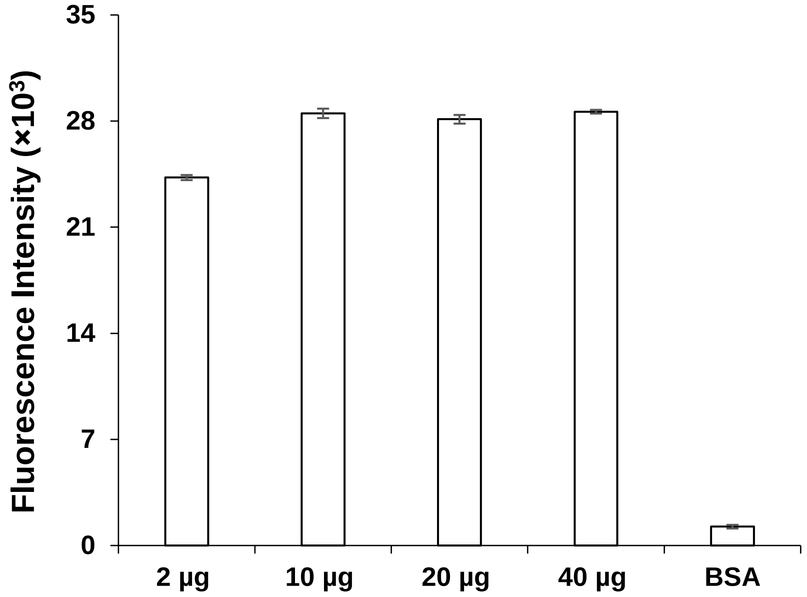

Figure S1. RCA-I binding results using MagPlex beads immobilized with different amounts of Lacβ-BSA. Different amounts of Lacβ-BSA with a valency of 36 glycan per BSA molecule were incubated with 25 µL (0.3 × 10^6 beads) of EDC/NHS-activated MagPlex beads (Region numbers 7, 8, 12, 13 for 2, 10, 20, 40 µg Lacβ-BSA respectively) at room temperature for 2 h. BSA (10 µg) immobilized (Region number 44) under the same condition was used as a control. RCA-I (15 µg/mL) was used in PBS+0.2% BSA as the binding buffer and PBS+0.1% Tween 20 was used as the washing buffer in the assay.

Glcα-BSA

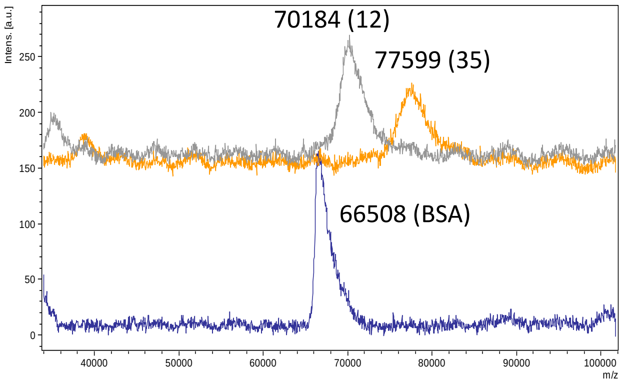

Glcβ-BSA

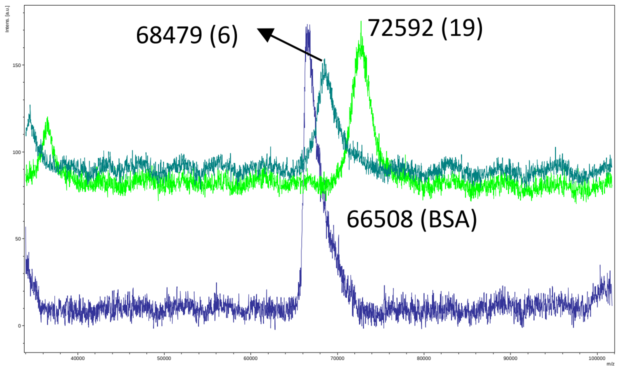

Galα-BSA

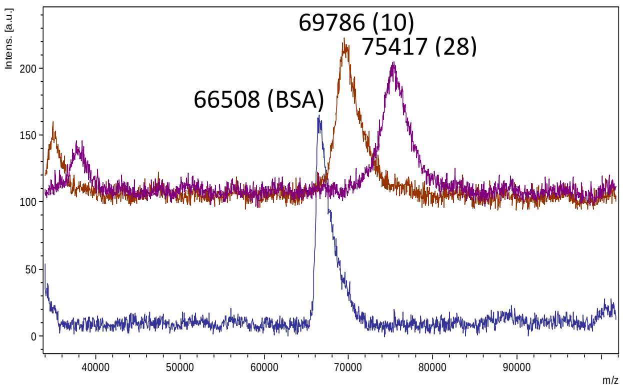

Galβ-BSA

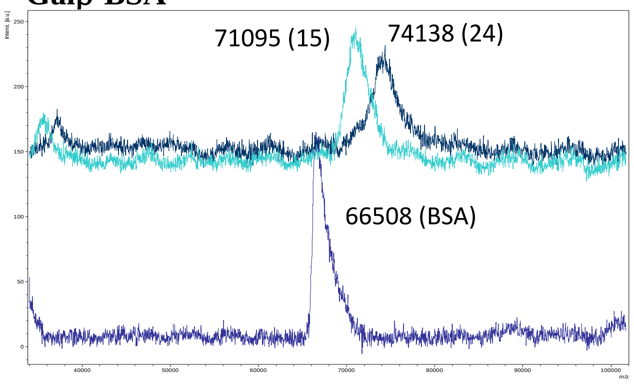

GlcNAcα-BSA

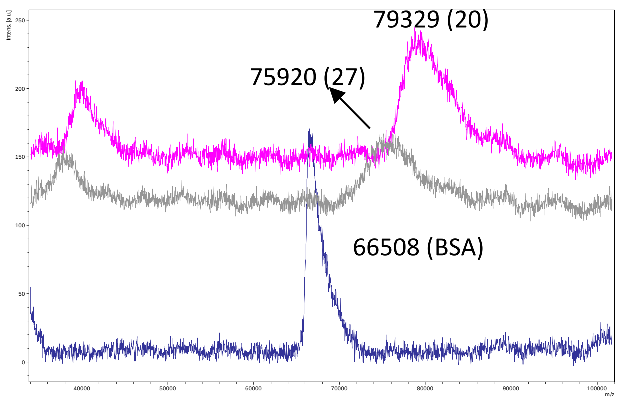

GlcNAcβ-BSA

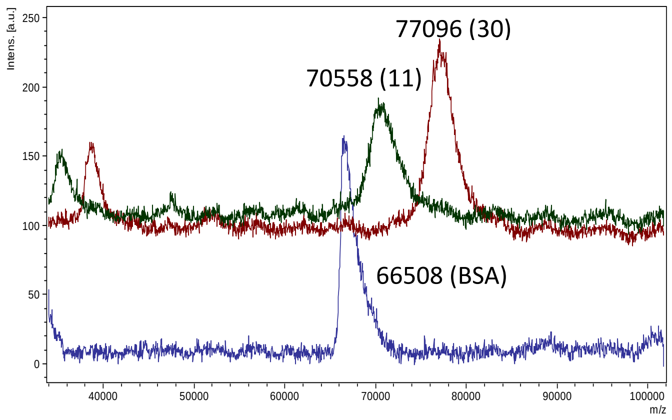

GalNAcα-BSA

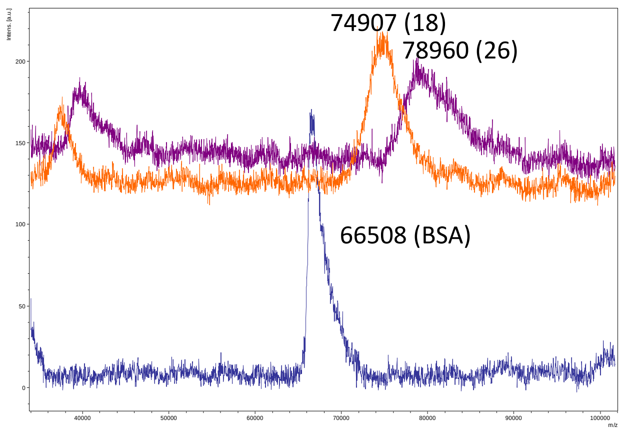

GalNAcβ-BSA

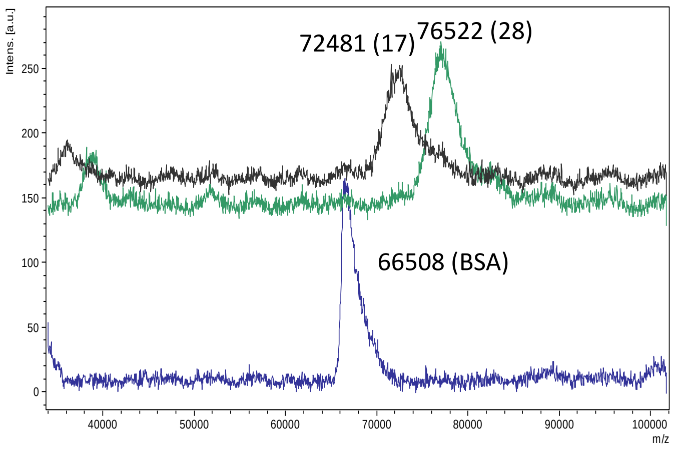

Galβ3GlcNAcα-BSA

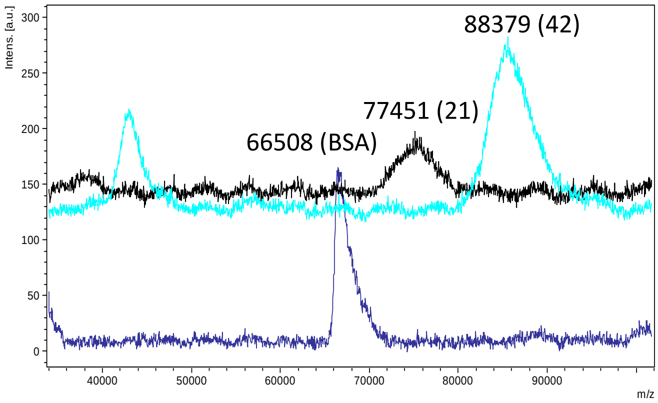

Galβ3GlcNAcβ-BSA

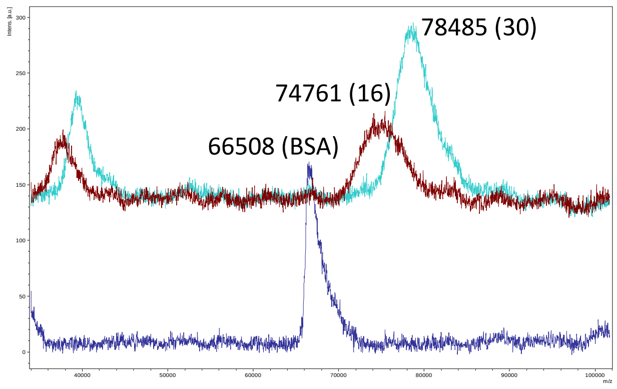

Galβ3GalNAcβ-BSA

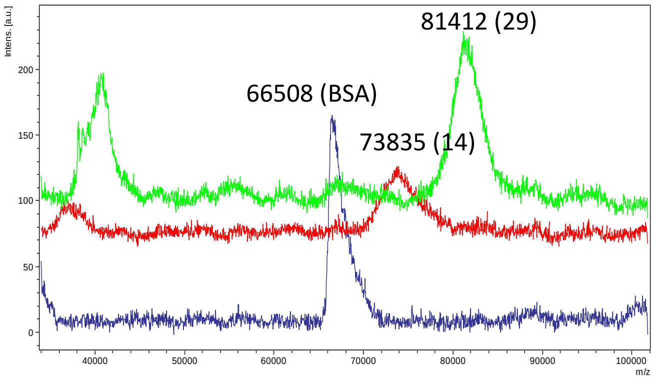

Galβ3GalNAcα-BSA

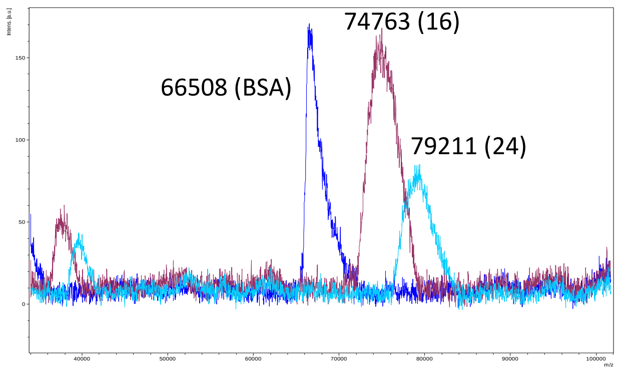

Lacβ-BSA

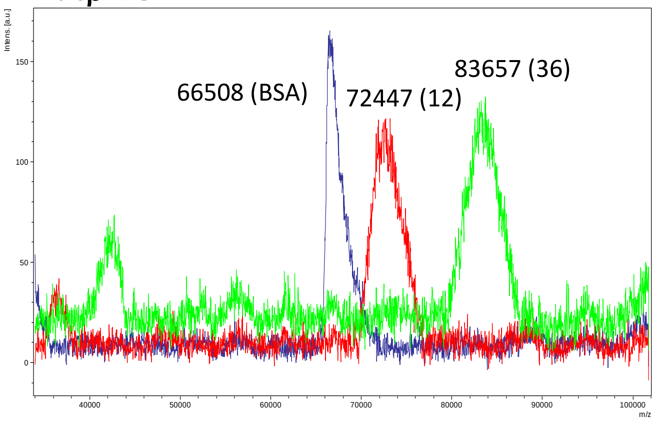

LacNAcβ-BSA

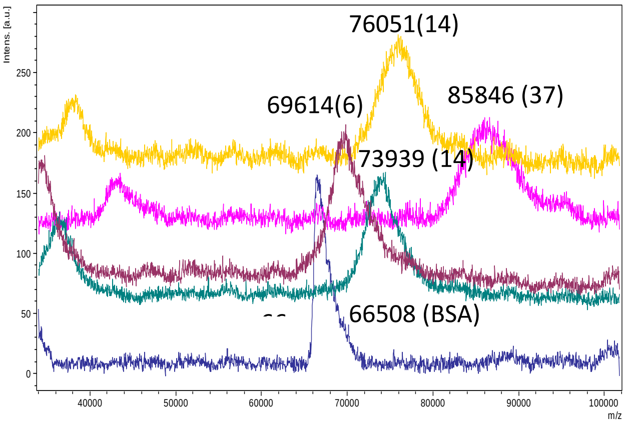

Galα3Lacβ-BSA (iGb3)

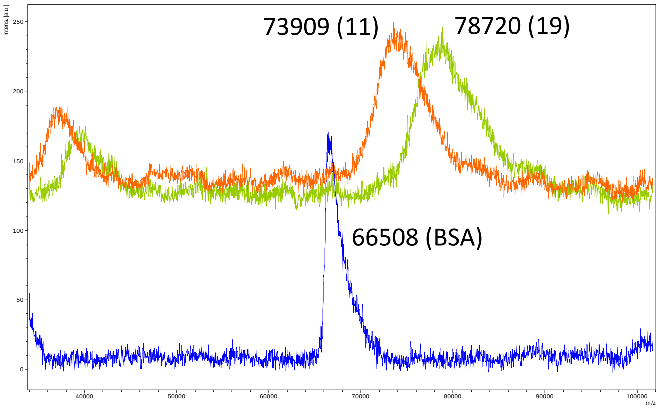

Galα3LacNAcβ-BSA

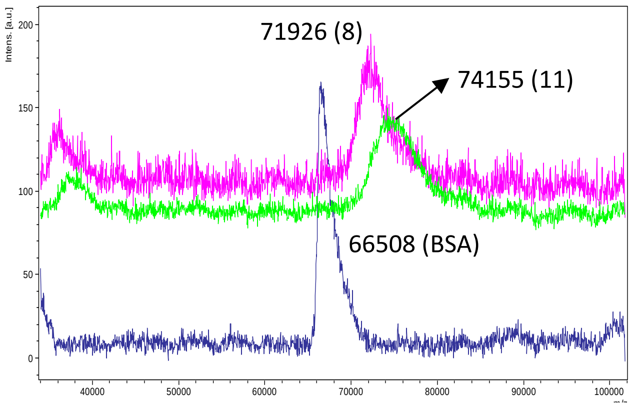

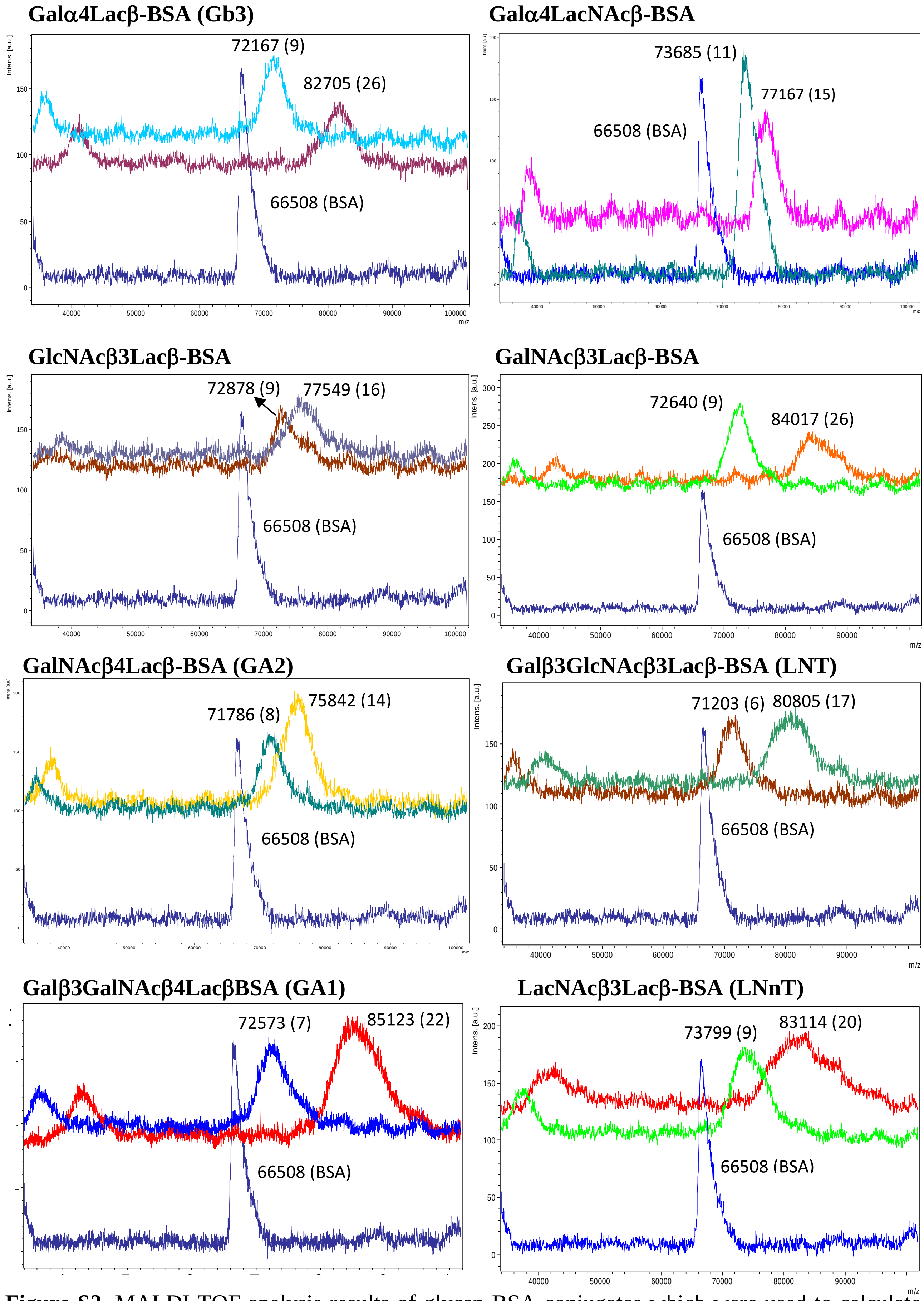

Figure S2. MALDI-TOF analysis results of glycan-BSA conjugates which were used to calculate the average numbers of glycans per BSA molecule (glycan valencies, the numbers shown in parentheses).

600 MHz ^{1}H and 150 MHz $^{13}C\{^{1}H\}$ NMR spectra of GlcαPropN$_3$ (**1**) in D_2O

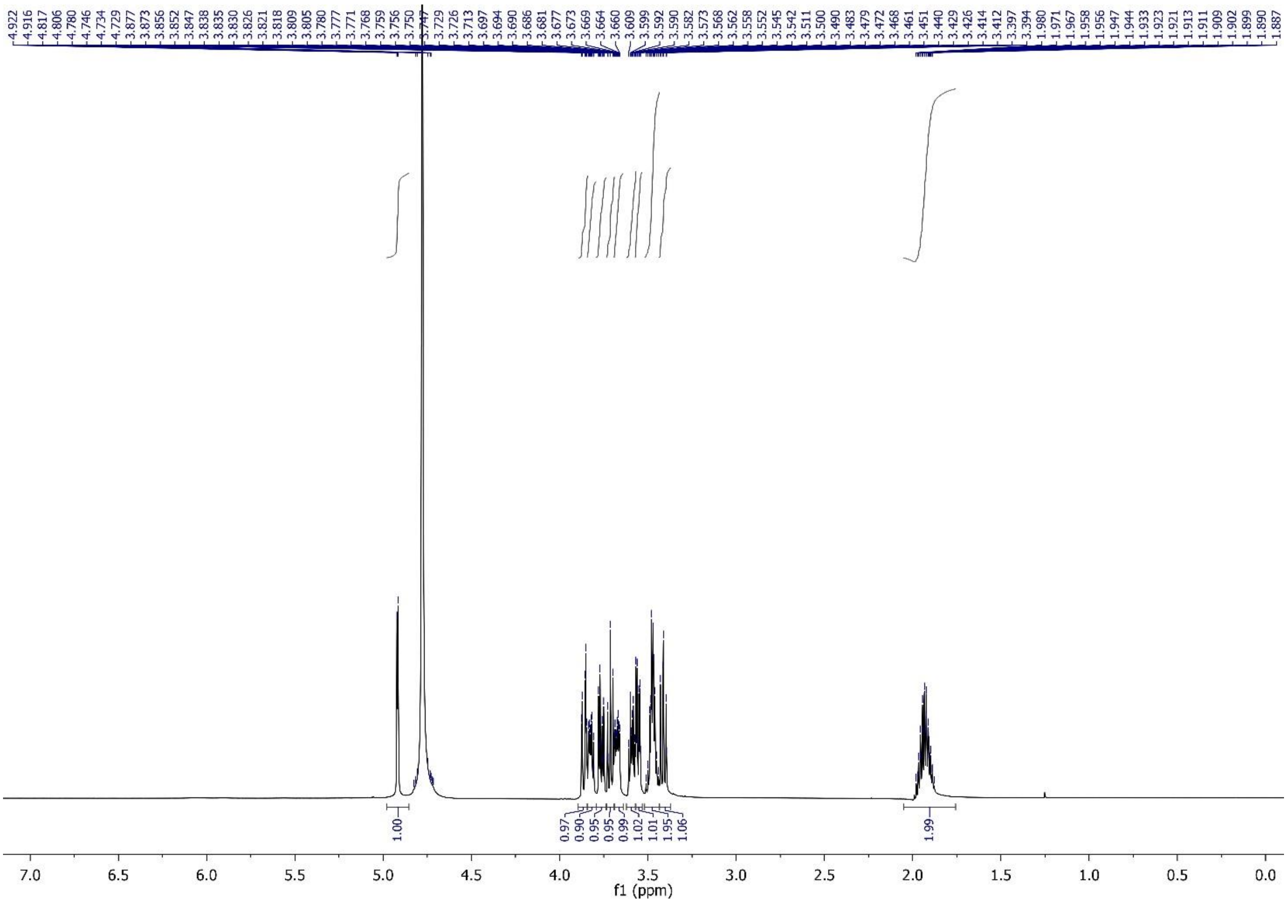

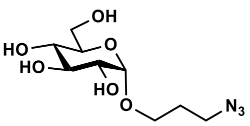

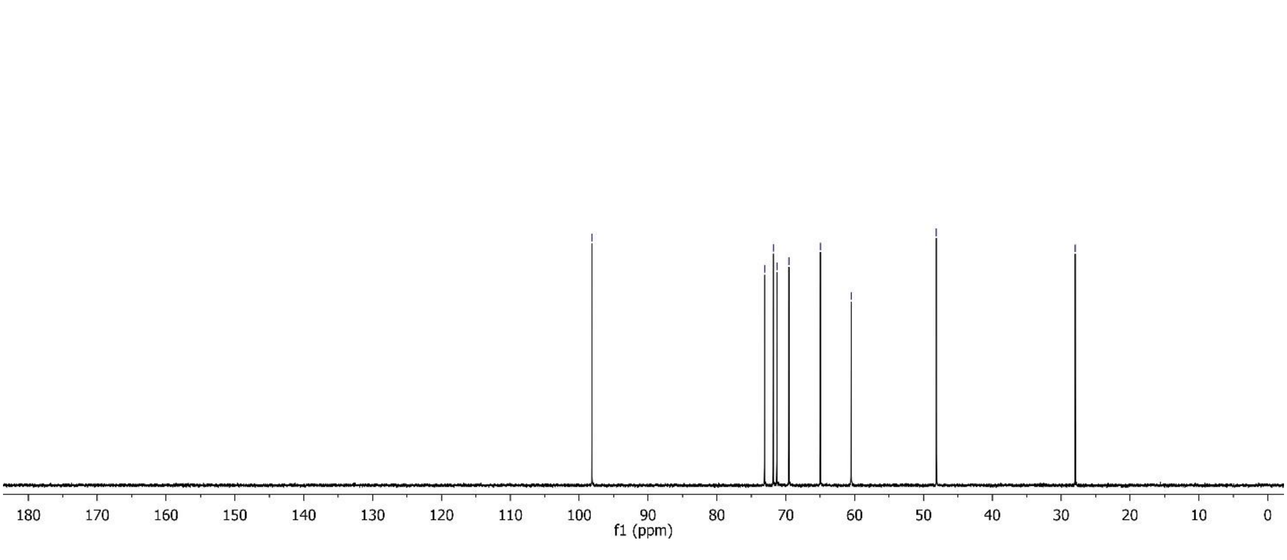

800 MHz ^{1}H and 200 MHz $^{13}C\{^{1}H\}$ NMR spectra of GlcβPropN$_3$ (**2**) in D_2O

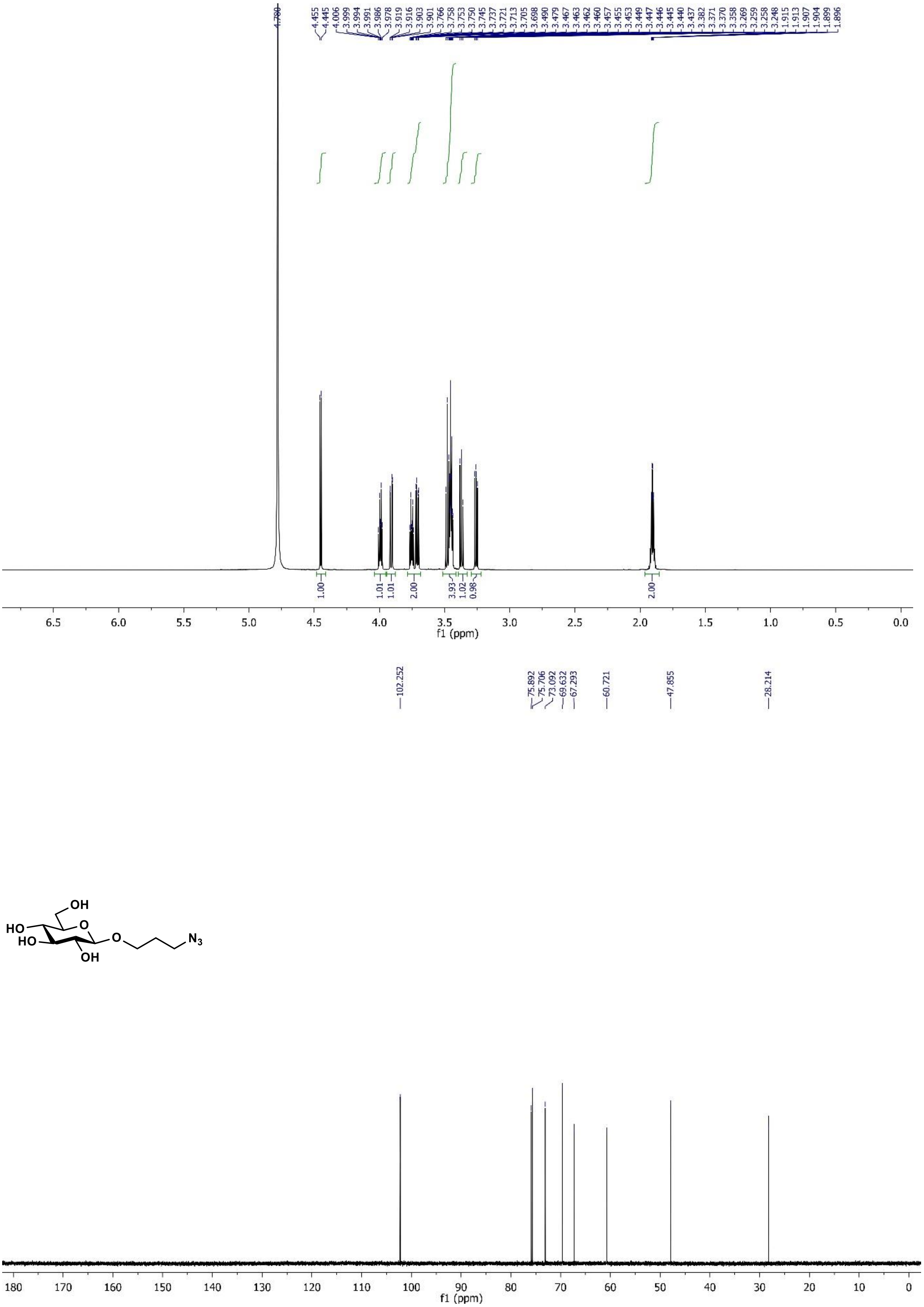

800 MHz ^{1}H and 200 MHz $^{13}C\{^{1}H\}$ NMR spectra of GalαPropN$_3$ (**3**) in D_2O

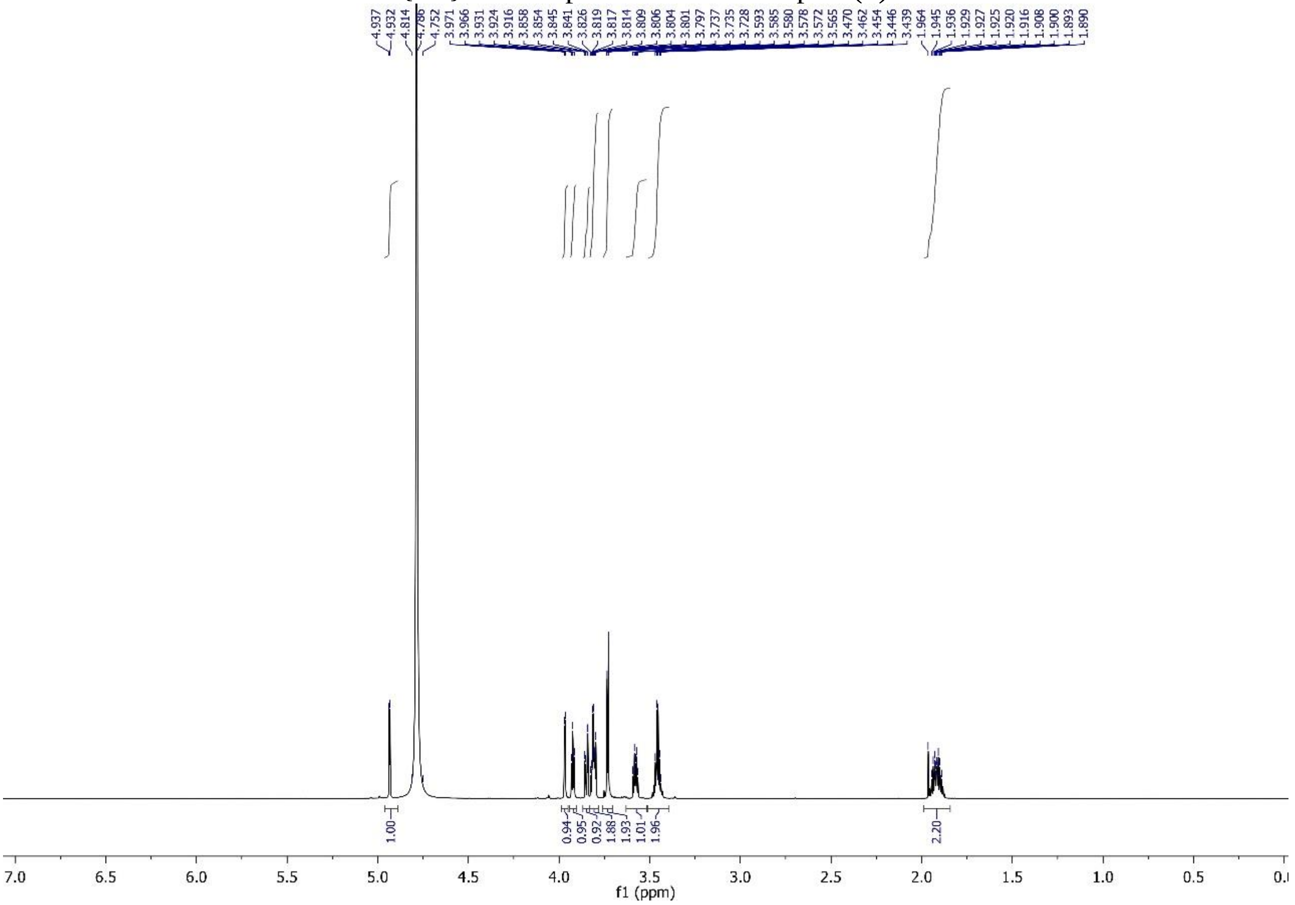

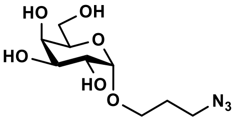

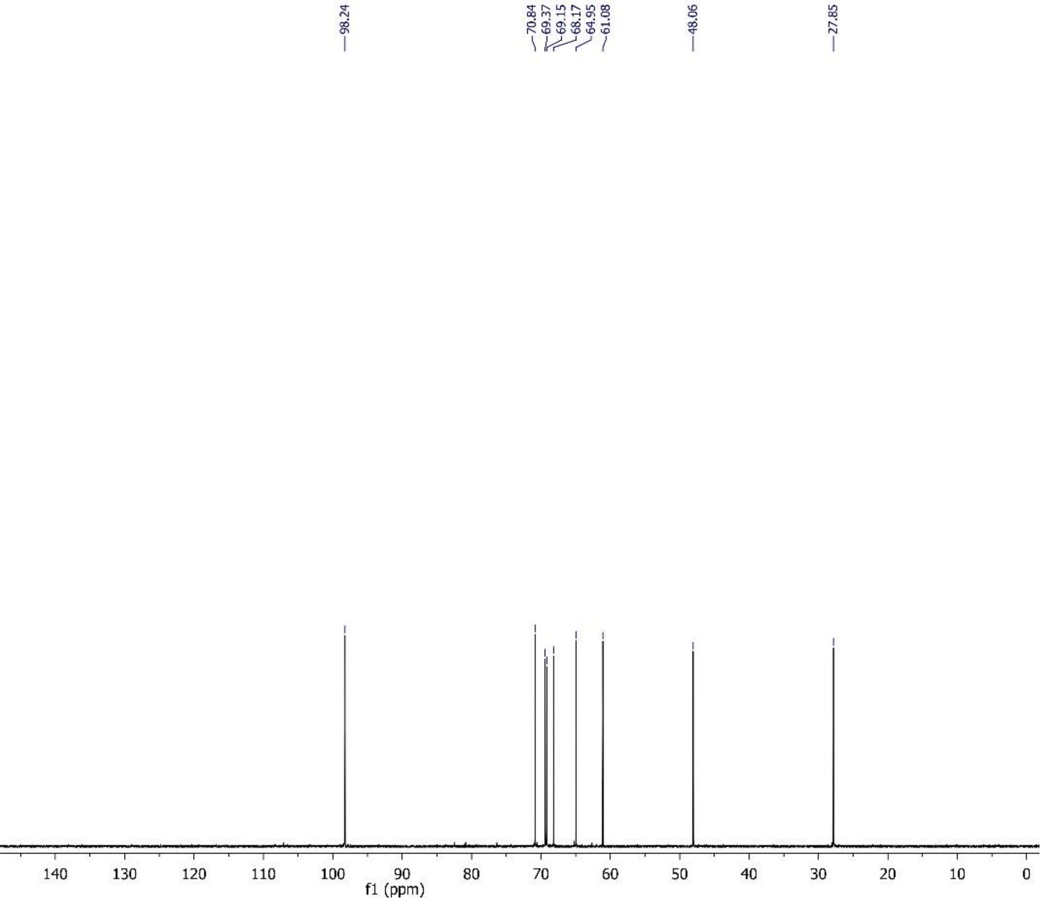

800 MHz ^{1}H and 200 MHz $^{13}C\{^{1}H\}$ NMR spectra of Galα3LacβPropN$_3$ (**15**) in D_2O

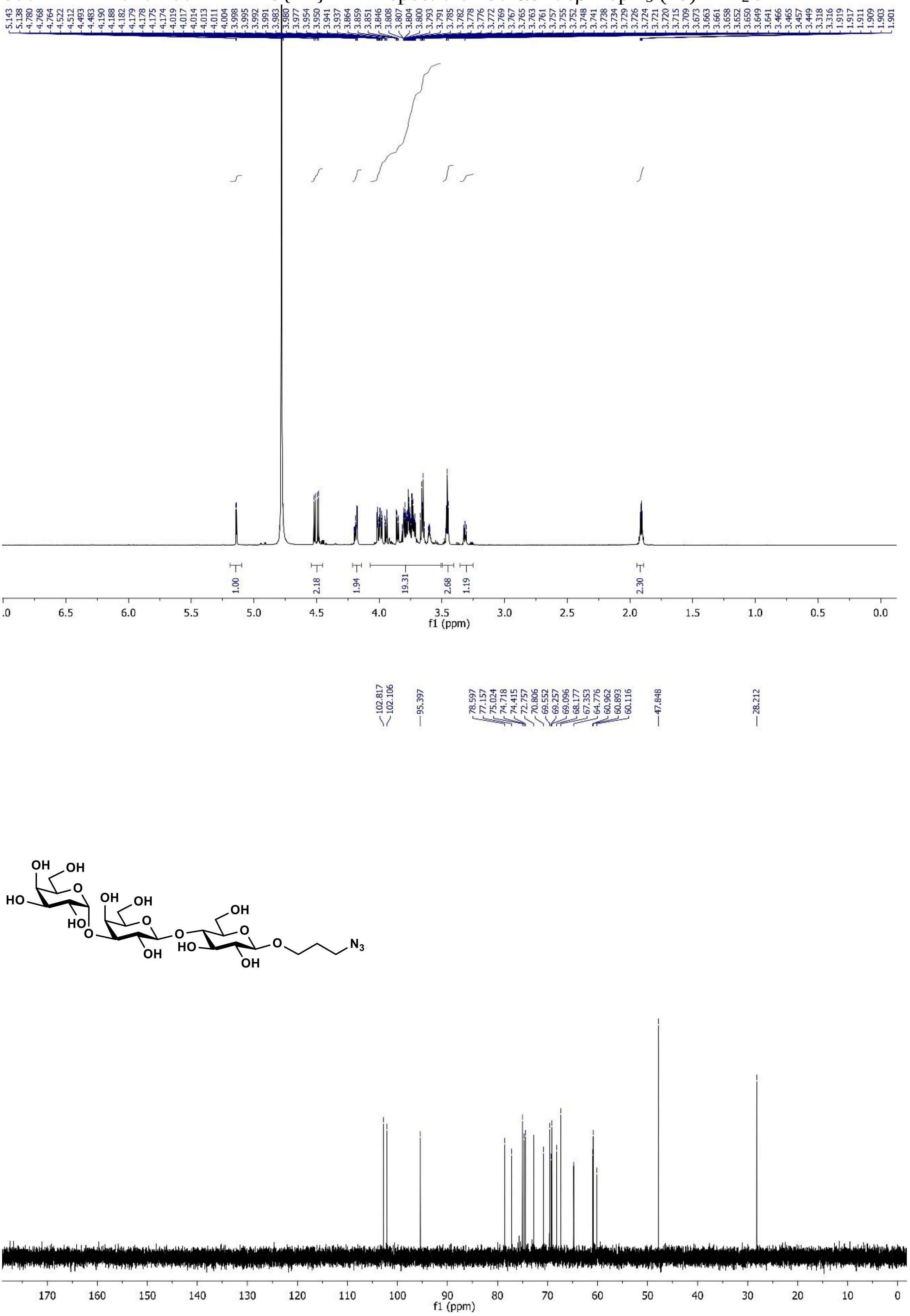

600 MHz ^{1}H and 150 MHz $^{13}C\{^{1}H\}$ NMR spectra of Galα3LacNAcβPropN$_3$ (**16**) in D_2O

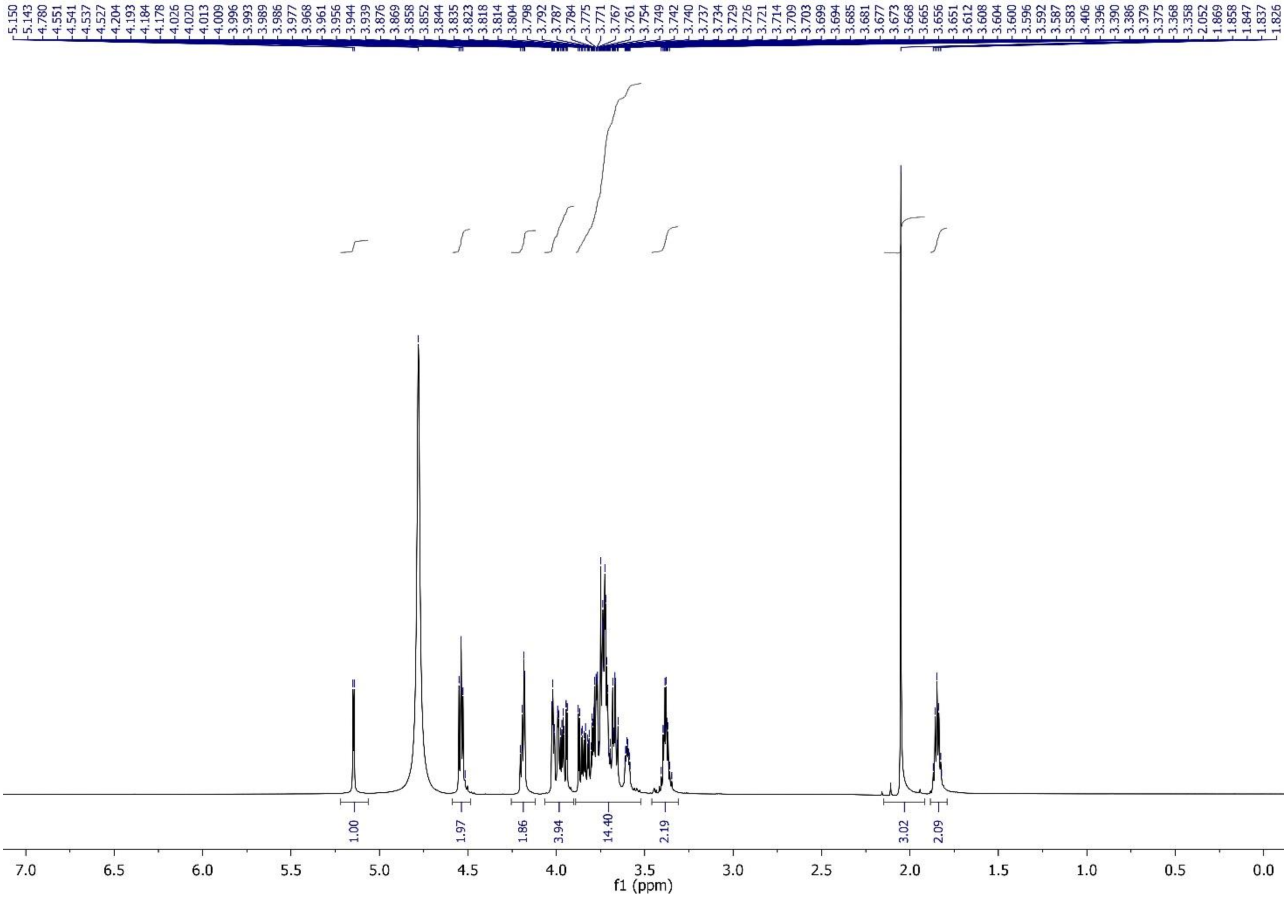

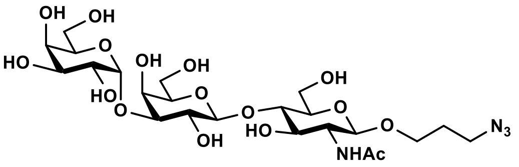

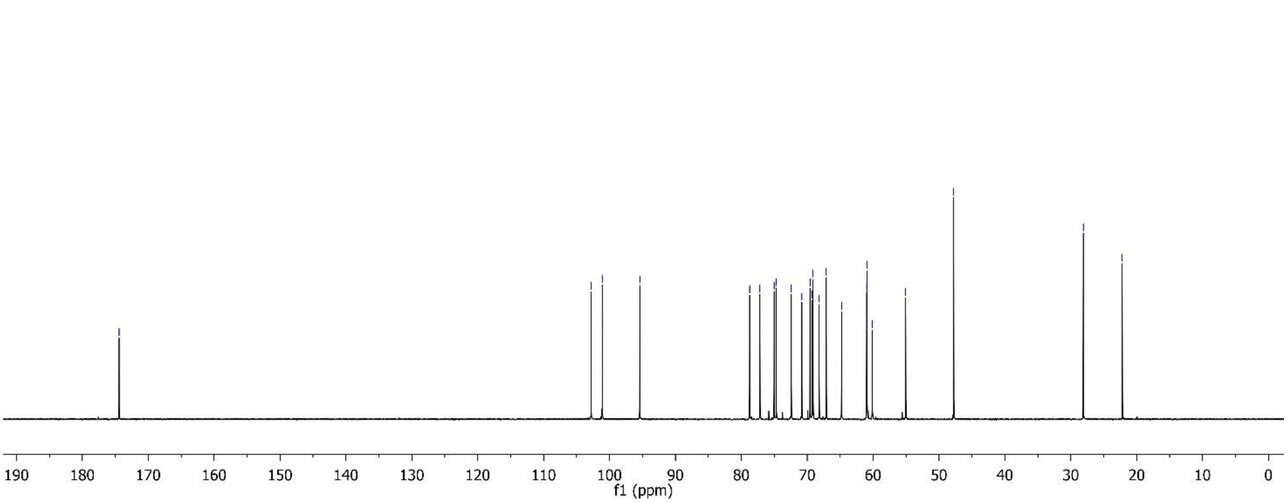

800 MHz ^{1}H and 200 MHz $^{13}C\{^{1}H\}$ NMR spectra of Galα4LacβPropN$_3$ (**17**) in D_2O

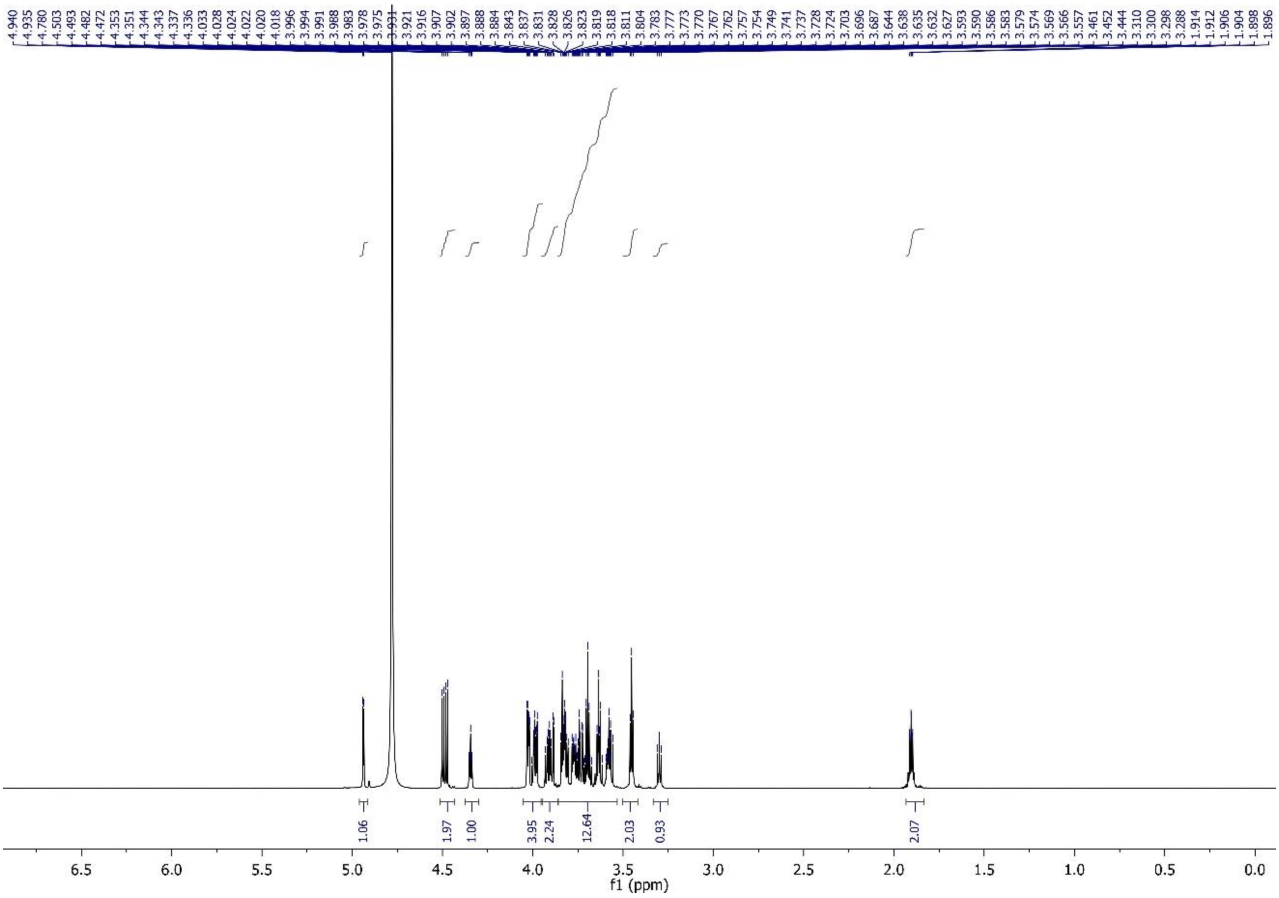

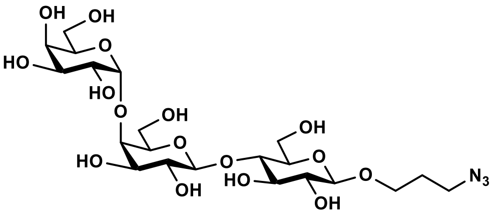

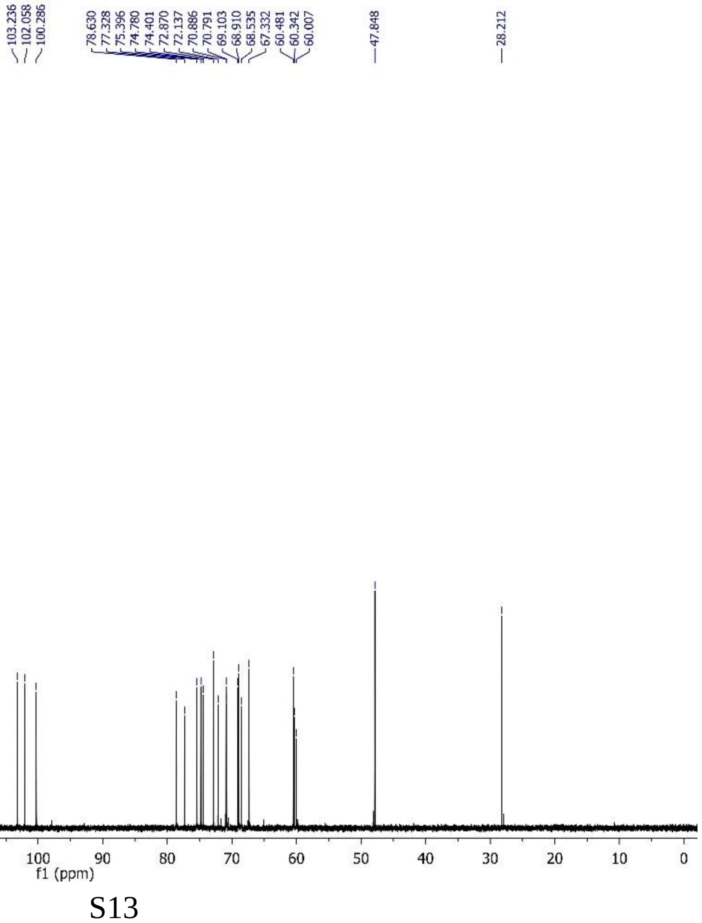

600 MHz ^{1}H and 150 MHz $^{13}C\{^{1}H\}$ NMR spectra of Galα4LacNAcβPropN$_3$ (**18**) in D_2O

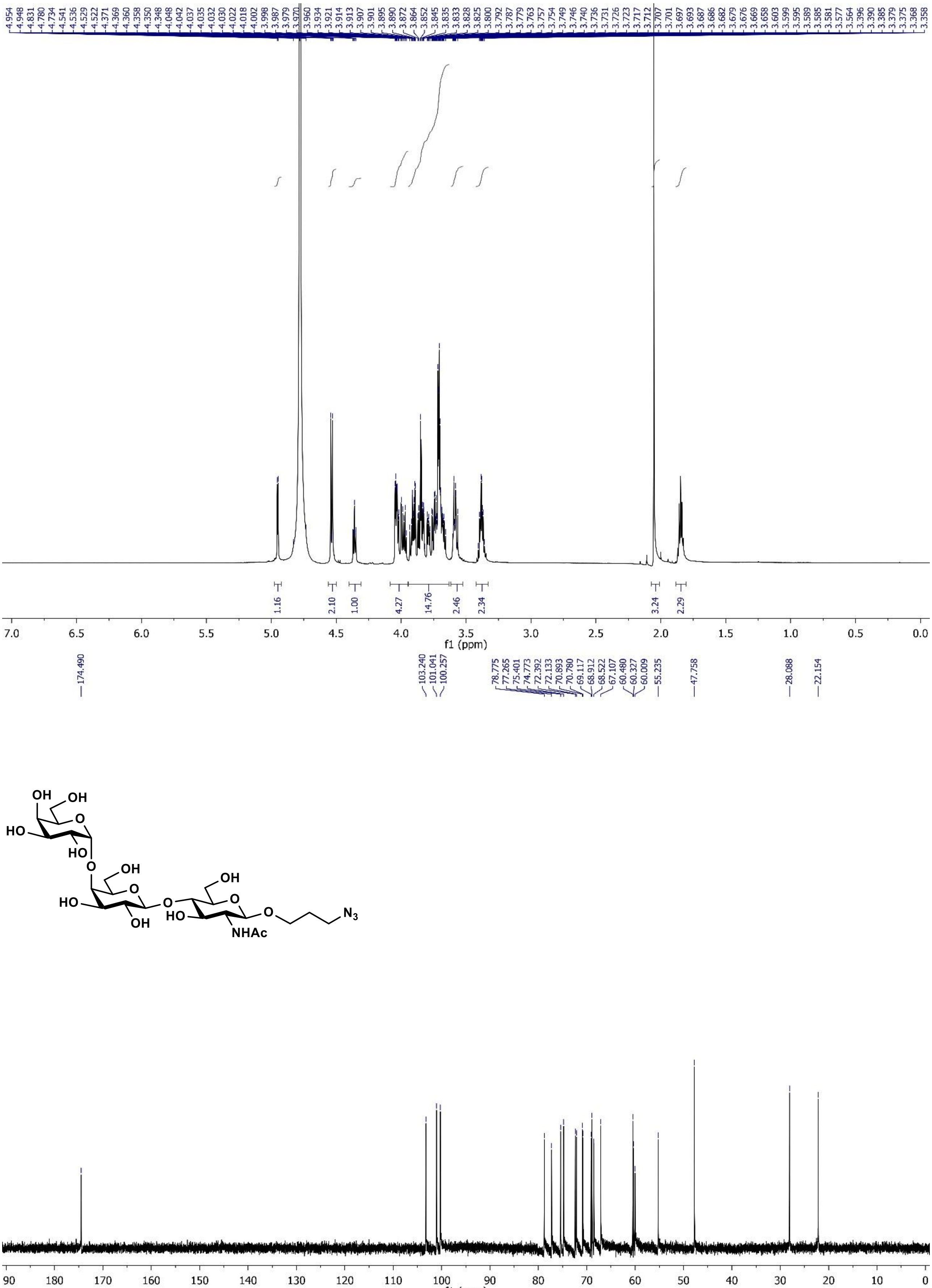

800 MHz ^{1}H and 200 MHz $^{13}C\{^{1}H\}$ NMR spectra of GalNAcβ3LacβPropN$_3$ (**20**) in D_2O

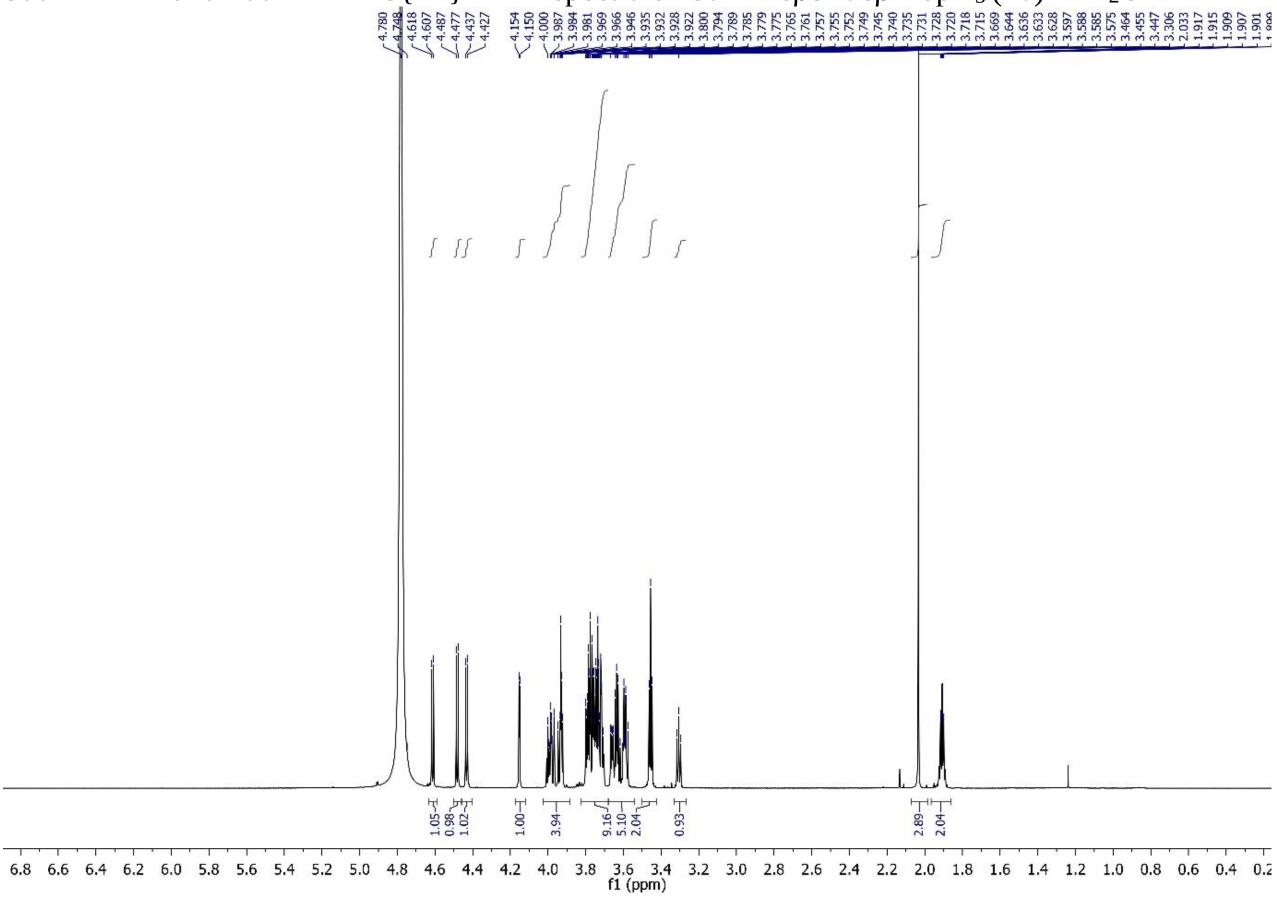

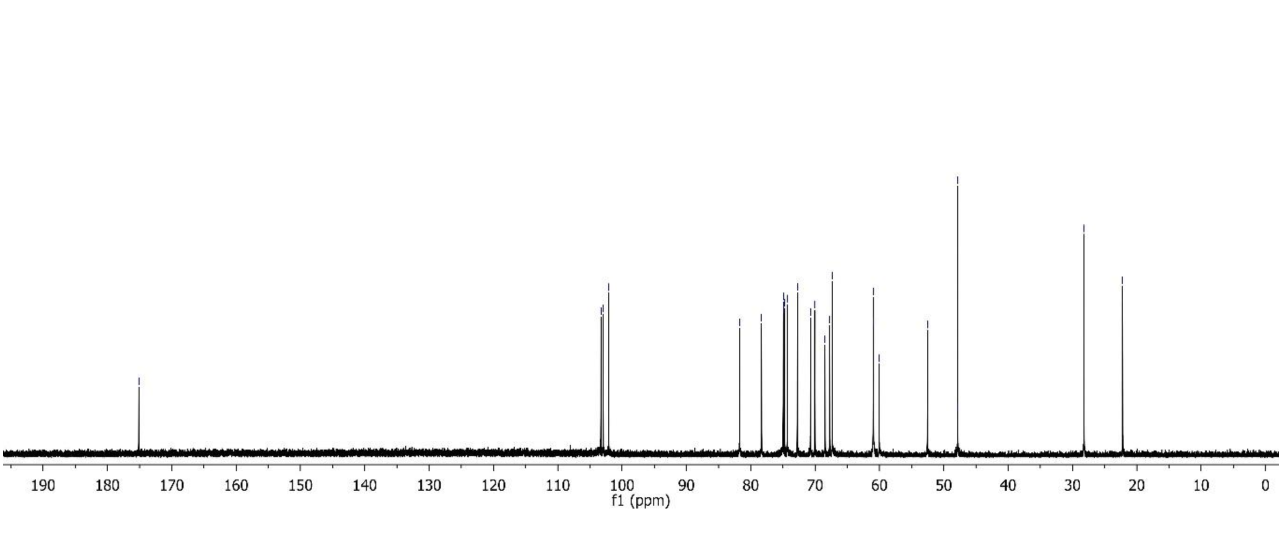

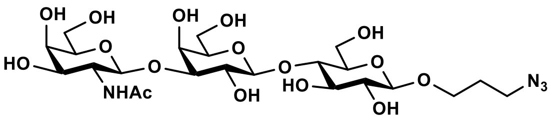

800 MHz ^{1}H and 200 MHz $^{13}C\{^{1}H\}$ NMR spectra of Galβ3GlcNAcβ3LacβPropN_3 (LNT, **22**) in D_2O

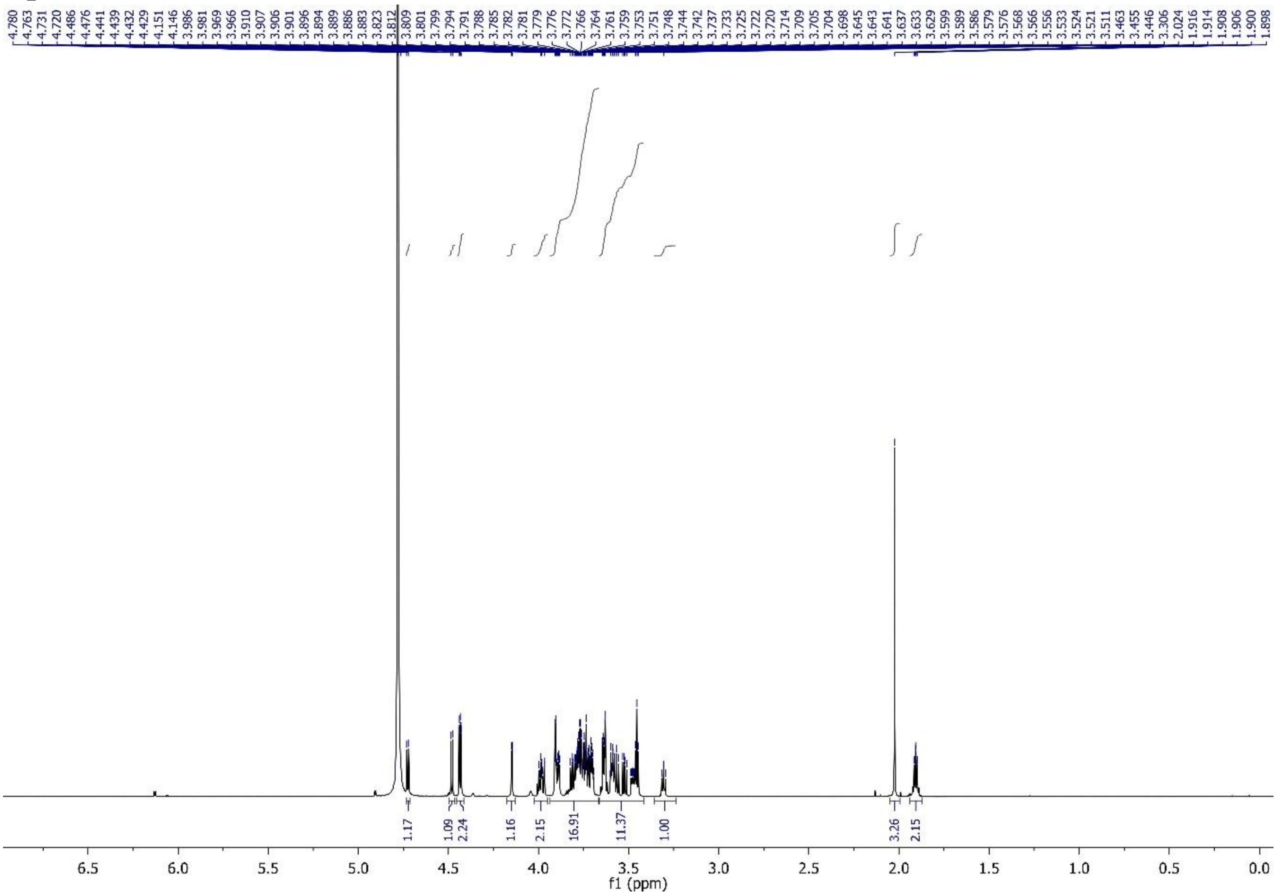

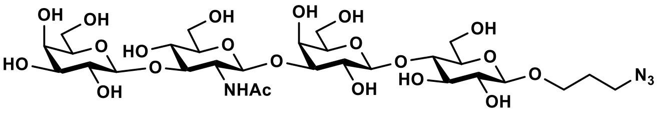

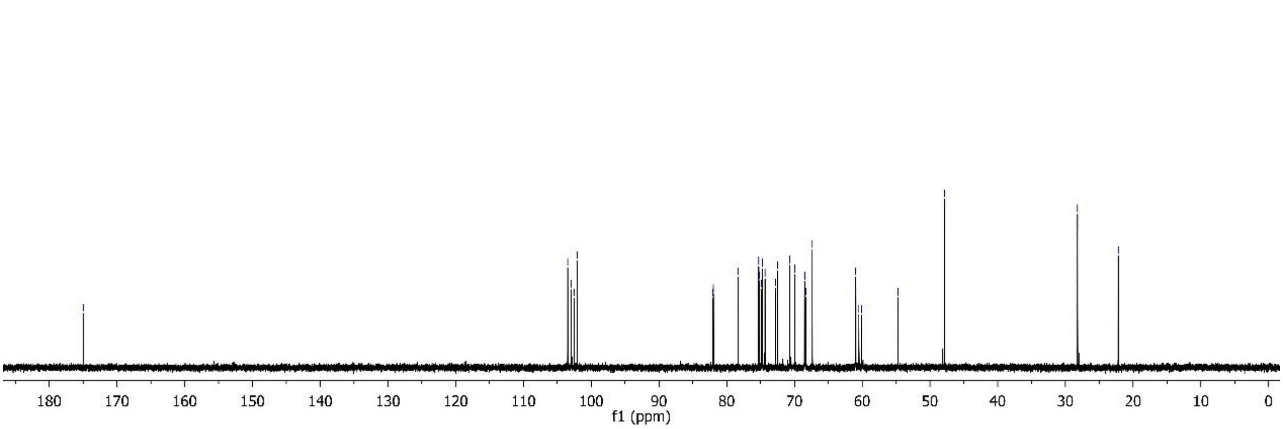

Supplement: Supplementary file 1 [file molecules-26-06194-s001.zip › molecules-1404673-supplementary.pdf]
